# Complete RNA inverse folding: computational design of functional hammerhead ribozymes

**DOI:** 10.1093/nar/gku740

**Published:** 2014-09-10

**Authors:** Ivan Dotu, Juan Antonio Garcia-Martin, Betty L. Slinger, Vinodh Mechery, Michelle M. Meyer, Peter Clote

**Affiliations:** 1 Biology Department, Boston College, 140 Commonwealth Avenue, Chestnut Hill, MA 02467, USA; 2 Hofstra North Shore-LIJ School of Medicine, Hempstead, NY 11549, USA

## Abstract

Nanotechnology and synthetic biology currently constitute one of the most innovative, interdisciplinary fields of research, poised to radically transform society in the 21st century. This paper concerns the synthetic design of ribonucleic acid molecules, using our recent algorithm, RNAiFold, which can determine all RNA sequences whose minimum free energy secondary structure is a user-specified target structure. Using RNAiFold, we design ten *cis*-cleaving hammerhead ribozymes, all of which are shown to be functional by a cleavage assay. We additionally use RNAiFold to design a functional *cis*-cleaving hammerhead as a modular unit of a synthetic larger RNA. Analysis of kinetics on this small set of hammerheads suggests that cleavage rate of computationally designed ribozymes may be correlated with positional entropy, ensemble defect, structural flexibility/rigidity and related measures. Artificial ribozymes have been designed in the past either manually or by SELEX (Systematic Evolution of Ligands by Exponential Enrichment); however, this appears to be the first purely computational design and experimental validation of novel functional ribozymes. RNAiFold is available at http://bioinformatics.bc.edu/clotelab/RNAiFold/.

## INTRODUCTION

Ribonucleic acid enzymes (a.k.a. ribozymes) are catalytic RNAs with enzymatic capabilities that, similar to their protein counterparts, can catalyze and accelerate the rate of biochemical reactions while maintaining a great specificity with respect to the substrate they act upon. In general, ribozymes can catalyze the transesterification of phosphodiester bonds, acting in *cis* by self-cleavage, or in *trans* by cleaving other RNAs. There exist different types of ribozymes, all with a very well defined tertiary structure: group I introns—self-splicing ribozymes, that were first observed for the intron of the nuclear 26S rRNA gene in *Tetrahymena thermophila* (1,2); group II introns—self-splicing ribozymes, which produce ligated exons and an excised intron-lariat as products of the splicing procedure (3); ribonuclease P (RNase P)—a ubiquitous endoribonuclease that processes the 5′ end of precursor tRNA molecules, producing 5′ phosphoester and 3′-OH termini (4) and small self-cleaving pathogenic RNAs, such as hammerhead ribozymes (5,6), as well as the hairpin and the hepatitis delta virus ribozymes (7).

### RNA synthetic biology

In response to the increased understanding and appreciation of the role RNA plays in biology, the last decade has seen a surge in the field of RNA synthetic biology. Several laboratories have successfully produced synthetic RNA sequences capable of self-cleaving, sensing small molecules *in vivo* or *in vitro*, as well as regulating gene expression (8,9). Many of these efforts have focused on the creation of allosteric ribozymes, or gene regulatory elements that can be used for further application.

Selection-based approaches (e.g. SELEX, or Systematic Evolution of Ligands by EXponential enrichment (10,11)) have proved very powerful for generating a range of RNAs with a variety of capabilities. Allosteric ribozymes that are inhibited or activated by specific small molecules have been achieved by utilizing a pre-existing self-cleaving ribozyme sequence coupled to either an existing aptamer (12), or one derived through selection (13). Additionally, SELEX has been coupled with *in vivo* screens to create RNAs with gene-regulatory activity in response to specific small molecule (14) or protein stimuli (15,16).

Design-based approaches have also been successful at creating RNAs with engineered functions. By a series of manually determined pointwise mutations, where biological activity was repeatedly assayed for intermediate structures, a single RNA sequence was designed to simultaneously support the catalytic activities of both the self-cleaving hepatitis delta virus ribozyme and the class III self-ligating ribozyme (17). Several approaches to designing genetic regulators mimic the action of small regulatory RNAs by introducing engineered trans-acting RNAs to occlude a ribosome binding site or start codon to inhibit translation. Gene expression may be altered in such systems by inhibiting the original RNA with a second trans-acting RNA (18), or through utilization of a ligand binding domain (aptamer) to induce an alternative RNA structure that does not interact with the transcript of interest (19). In addition, hammerhead ribozymes have been used to target the HIV virus (20,21) by modifying sequences within base-pairing regions to target a specific sequence of viral RNA.

As the complexity of synthetic RNA devices increases, there is an increasing need to go beyond *ad hoc* manual approaches, and *in vitro* selection methods. RNA molecules have been rationally designed by the assembly of structural RNA tertiary fragments/motifs, extracted from X-ray and nuclear magnetic resonance structures of natural RNA molecules (22,23); see also (24). Using computational methods with *reaction graphs*, with subsequent validation using atomic force microscopy, molecular programs have been executed for a variety of dynamic DNA constructs, ranging from hairpins, binary molecular *trees*, to bipedal walkers (25). RNA thermoswitches have been computationally designed and synthesized, that are as efficient as natural thermoswitches, by applying the program, switch.pl (26), which attempts to minimize the following cost function for input RNA sequence **a** = *a*_1_, …, *a*_*n*_:

\begin{eqnarray*} &&(E_{T_1}({\bf a},S_1)-G_{T_1}({\bf a}) ) +( E_{T_2}({\bf a},S_2) -G_{T_2}({\bf a}) )\\ &&-\xi ((E_{T_1}({\bf a},S_1)-E_{T_1}({\bf a},S_2))\\ &&+ (E_{T_2}({\bf a},S_2)-E_{T_2}({\bf a},S_2)) \end{eqnarray*}

where *G*_*T*_(**a**) is the ensemble free energy sequence **a** at temperature *T*, *E*_*T*_(**a**, *S*) is the free energy of RNA sequence **a** with structure *S* at temperature *T*, and 0 < ξ < 1 is a constant. Waldminghaus et al. (27) selected promising thermoswitch candidate sequences returned by switch.pl by considering the cost function values, the predicted melting temperature (RNAheat (28)) etc. The resulting candidates were not functional; however, functional thermoswitches were obtained from these candidates after several rounds of error-prone polymerase chain reaction (PCR) mutagenesis and *in* *vitro* selection. Recently, a synthetic theophylline riboswitch has been rationally designed to *transcriptionally* regulate the expression of a gene, by fusing a theophylline aptamer with a computationally designed expression platform (29). However, to the best of our knowledge, no group has previously designed a ribozyme by purely computational means, using RNA inverse folding, and subsequently validated the ribozyme functionality; this is our contribution in the present article.

### Molecular design and RNA inverse folding

Given an RNA sequence, the *folding* problem is to determine the *native structure* into which the sequence folds; in contrast, given a target RNA structure, the *inverse folding* problem is to determine one, several, or all sequences whose native structure is the given target structure. Since the pioneering work of Anfinsen (30), it is widely accepted that the native structure of a given macromolecule can be identified with its minimum free energy (MFE) structure. If we identify native structure with the MFE tertiary structure, then both the folding and inverse folding problems are NP-complete (31,32). However, since RNA secondary structure appears to form prior to tertiary interactions, thus creating a scaffold for tertiary structure formation (33,34), and since the folding and inverse folding problems are intractable for tertiary structures, we consider the folding and inverse folding problems for RNA secondary structure in this paper.

Using free energy parameters obtained from optical melting experiments (35), the dynamic programming algorithm of Zuker (36) determines the MFE secondary structure of a given RNA in cubic time. This algorithm has been implemented in (28,37–41), where it should be noted that secondary structure predictions may differ due to different treatment of dangles, coaxial stacking, etc. and their corresponding energy parameters.

It seems likely that inverse folding is NP-complete, even for RNA secondary structures (42); nevertheless, a number of heuristic algorithms exist that return approximate solutions: RNAinverse (43), switch.pl (26), RNA-SSD (44), INFO-RNA (45), MODENA (46), NUPACK-DESIGN (47), Inv (48), Frnakenstein (49). Rather than employing a heuristic, our recent software, RNAiFold (50,51), employs Constraint Programming (CP) (52), which always returns exact solutions, although it might do so in an impractical amount of time. Moreover, CP is the only inverse folding software capable of determining whether (provably) no solution exists—i.e. that no RNA sequence has MFE secondary structure that is identical to the target structure. Additionally, CP allows us to model and account for several RNA sequence design constraints that are necessary for a more biologically relevant result—for instance, controlling GC content, describing fixed upper and lower bounds for certain types of base pairs, limiting a maximum number of consecutive nucleotides of a given type, specifying certain mononucleotide and/or dinucleotide frequencies, requiring specific nucleotides that are suspected to constitute the active site, etc. CP can also enforce *compatibility* constraints and *incompatibility* constraints, which require that all returned sequences not only fold into the given target structure, but additionally are compatible (incompatible) with another user-stipulated structure.

## MATERIALS AND METHODS

### Computational methods

RNAiFold returns sequences whose MFE structure is a given target structure, whereby the user may choose to use the free energy parameters from either Vienna RNA Package 1.8.5 (Turner 1999 parameters) or Vienna RNA Package 2.0.7 (Turner 2004 parameters) (53). By abuse of notation, let RNAiFold 1.8.5 [resp. 2.0.7] denote the program RNAiFold with energy parameters from the corresponding version of Vienna RNA Package.

As target structure for our computationally designed type III hammerheads, we selected the secondary structure of a portion of the plus polarity strand of Peach Latent Mosaic Viroid (PLMVd) (isolate LS35, variant ls16b) from Rfam family RF00008 (54) having accession code AJ005312.1/282-335. The reason we chose PLMVd AJ005312.1/282-335 was that this is the only RNA sequence in the seed alignment of RF00008, whose MFE structure is identical to its Rfam consensus structure, when computed by RNAfold 1.8.5—see Supplementary Information for a precise definition of Rfam consensus structure. Moreover, as shown in Supplementary Information Figure S1, the MFE structure computed by RNAfold 2.0.7 differs markedly from the Rfam consensus structure of PLMVd AJ005312.1/282-335, hence we used RNAiFold with the energy parameters from Vienna RNA Package 1.8.5. In summary, the target structure for RNAiFold 1.8.5 was taken to be

\begin{equation*} .((((((.(((((...))))).......((((........))))...)))))). \end{equation*}

which is both the RNAfold 1.8.5 MFE structure as well as the Rfam consensus structure of PLMVd AJ005312.1/282-335.

**Figure 1.**
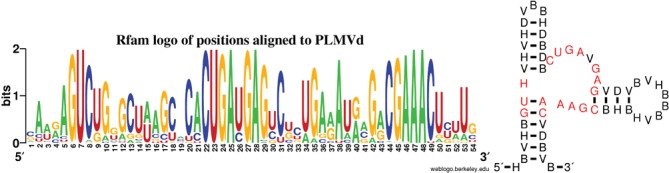
(Left) Sequence conservation for the 56 nt consensus sequence for type III hammerhead ribozymes from version 11.0 of the Rfam database (54); image from http://rfam.sanger.ac.uk/family/RF00008#tabview=tab3. (Left) Sequence logo of conservation at positions aligned with the 54 nt Peach Latent Mosaic Viroid (PLMVd) AJ005312.1/282-335 from the hammerhead ribozyme type III seed alignment sequences from Rfam family RF00008. In-house program used to determine frequencies of positions aligned to those of PLMVd; sequence logo generated with WebLogo (64) (web server at http://weblogo.berkeley.edu/). The 15 positions 6–7, 22–25,27–29, 44–49 of PLMVd had sequence conservation in excess of 96%, while cleavage site C at position 8, adjacent to region 6–8, was conserved in 94.9367% of RF00008 seed alignment sequences. RNAiFold was subsequently used to solve the inverse folding problem with consensus structure of PLMVd used as target, with sequence constraints at positions 6–8, 22–25, 27–29, 44–49, as explained in text. Resulting from this analysis, the sequence constraints for RNAiFold were defined to be HBVHBGUHVH VHDVBBHDBD BCUGAVGAGV DVBVHBBBVH BHBCGAAACV DBVB. (Right) Sequence constraints for RNAiFold with indicated target secondary structure. The 15 positions 6-7, 22–25,27–29, 44-49 having over 96% sequence conservation in the seed alignment of RF00008 were constrained to be those in PLMVd AJ005312.1/282-335, and the cleavage site 8 was constrained to be H (not G). All 38 remaining positions were constrained to be distinct from the corresponding nucleotides in PLMVd.

Numerous biochemical and structural studies have pinpointed key nucleotides in the hammerhead ribozome that are required for catalysis (55–57). However for an efficient, purely computational design of synthetic hammerheads, it is important to rely only on sequence conservation results from reliable multiple alignments. The Rfam web site image http://rfam.sanger.ac.uk/family/RF00008#tabview=tab3 clearly shows certain regions of the 56 nt consensus sequence have highly conserved sequence identity. Based on this observation, we computed the nucleotide frequency for the seed alignment of Rfam family RF00008 for those positions aligned to the nucleotides of the 54 nt PLMVd AJ005312.1/282-335. Figure 1 (left) shows the sequence logo of positions aligned to PLMVd AJ005312.1/282-335. Supplementary Information Table 1 shows that sequence identity exceeds 96% for the 15 positions 6–7, 22–25,27–29, 44–49 of PLMVd in the seed alignment for Rfam family RF00008 consisting of 84 sequences. For that reason, the nucleotides in PLMVd at these 15 positions were provided as a constraint for RNAiFold, thus fixing ∼28% of the 54 nucleotides. Note that the cleavage site at C8, discussed below would have been included in the constraints, had we chosen to retain positions of at least 95%.

**Table 1.** Hammerhead candidates selected and selection criteria used

ID	Sequence	Selection criteria
HH1	UUAAUGUAGAGCGAUUCGUUCCUGAAGAGCUAUAAUUUCUUAGCGAAACAUUAU	GC-content 30−39%, *P*(*S*_0_, **s**) ≥ 40%, smallest (binary) entropy distance for conserved site
HH2	UUAUUGUAGCGCGAUUCGCGCCUGAAGAGAUGCGUUUUAACAUCGAAACAGUAU	GC-content 40−49%, *P*(*S*_0_, **s**) ≥ 40%, smallest (binary) entropy distance for conserved site
HH3	CUAUUGUAGCGCGAUUCGCGCCUGAAGAGAUCUGUUUUAUGAUCGAAACAGUAU	GC-content 40−49%, *P*(*S*_0_, **s**) ≥ 40%, second smallest (binary) entropy distance for conserved site
HH4	UGGAUGUAGCGCGAUUCGCGCCUGAAGAGCGGUCAUCCAUCCGCGAAACAUUCU	GC-content 50−59%, *P*(*S*_0_, **s**) ≥ 40%, smallest (binary) entropy distance for conserved site
HH5	CUCAGGUAGCGCGAUUCGCGCCUGAGGAGGGGUCUGGUAUCCCCGAAACCUGAU	GC-content 60−69%, *P*(*S*_0_, **s**) ≥ 40%, smallest (binary) entropy distance for conserved site
HH6	UGGCGGUAGCGCGAUUCGCGCCUGAAGAGGGGUAACGCGUCCCCGAAACCGUCU	GC-content 30−39%, *P*(*S*_0_, **s**) ≥ 40%, *largest* (binary) entropy distance for conserved site
HH7	UCAAUGUCGCGCGAUUCGCGCCUGAAGAGAUGGAAUUUAACAUCGAAACAUUGU	GUC in positions 6–8, smallest ensemble defect
HH8	UCAAUGUAGCGCGAUUCGCGCCUGAAGAGAUGGAAUUUAACAUCGAAACAUUGU	smallest ensemble defect
HH9	UUAAUGUCGCGCGAUUCGCGCCUGAAGAGAUCUGACUUCUGAUCGAAACAUUAU	*P*(*S*_0_, **s**) ≤ 20%, smallest (binary) entropy distance for conserved site
HH10	UUAAGGUCGCGCGAUUCGCGCCUGACGAGCUAUAUUUUAUUAGCGAAACCUUAU	smallest (binary) entropy distance for conserved site

Note that, subject to presence or absence of additional constraint C8, HH7 and HH8 had also the largest probability of structure, the smallest full structural positional entropy, the smallest (Morgan-Higgs and Vienna) structural diversity and smallest expected base pair distance.

From the literature, it is well-known that hammerhead cleavage sites are of the form NUH (e.g. GUH and CUH); see, for instance, papers of Pan et al. (58) and Gonzalez-Carmona et al. (59), which provide experimental data on the efficiency of various target hammerhead cleavage sites. For PLMVd, cleavage occurs immediately after the cytidine at position 8. For this reason, IUPAC code H (i.e. not G) was given as an additional constraint at position 8 for RNAiFold.

**Figure 2.**
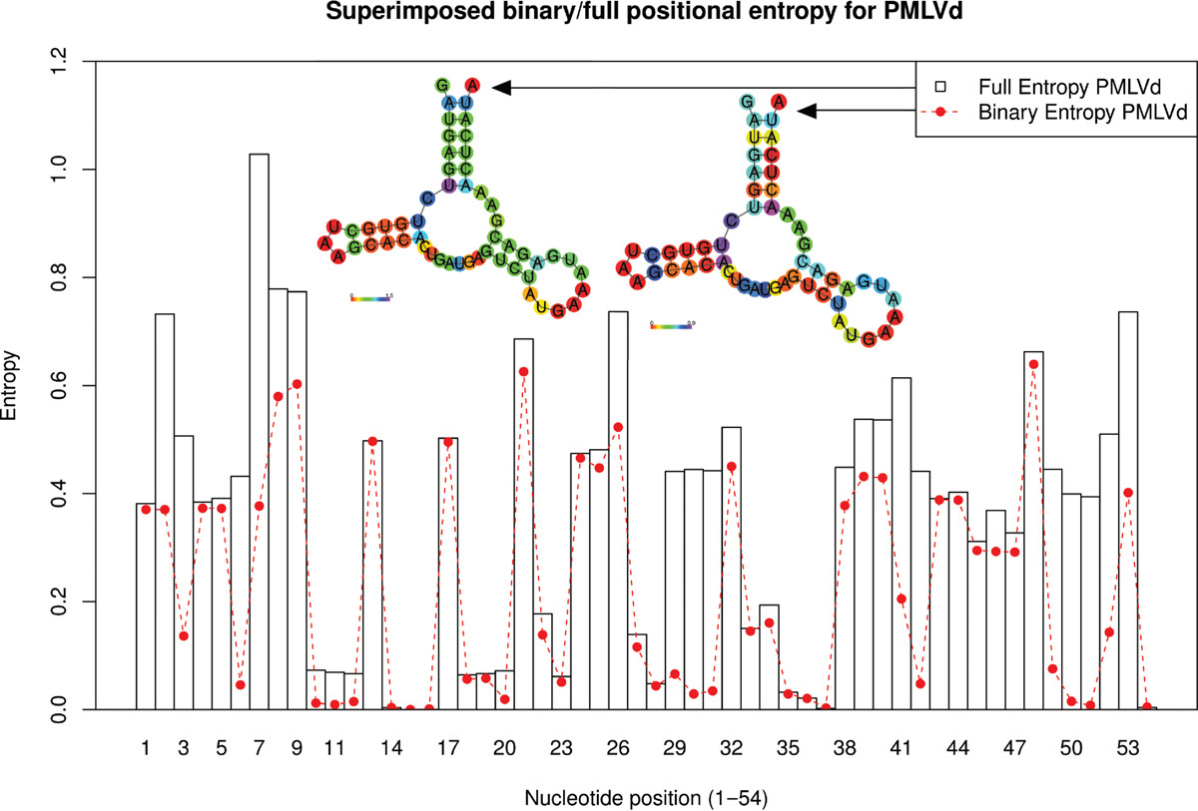
Binary and full structural positional entropy of hammerhead Peach Latent Mosaic Viroid (PLMVd) AJ005312.1/282-335. (Left) Full structural positional entropy *H*. (Right) Binary structural positional entropy *H*_*b*_. Note that positions 50, 51 of have medium (full) entropy and high binary entropy, which indicates that these positions tend always to be base-paired in the low energy ensemble of structures, though with different base pairing partners. Note that the conserved region GUH in 6–8 has moderate to high entropy (G6: 0.62, U7: 1.48, H8: 1.12), GUC in 22–24 has low entropy (G22: 0.26, U23: 0.09, C24: 0.68), GAG in 27-29 has low entropy (binary entropy is very low) (G27: 0.12, A28: 0.04, G29: 0.07), while 44-49 has medium entropy. Left colored secondary structure figure created using relplot.pl from Vienna RNA Package (28); right upper colored secondary structure figure created by modifying code relplot.pl.

Apart from nucleotide constraints at positions 6–7, 22–25, 27–29, 44–49, and the constraint H8, all nucleotides at the remaining 38 positions were constrained to be *distinct* from those of PLMVd—this was done to prevent any unintentional use of other nucleotide identities in the computational design of a hammerhead. Summarizing, each sequence returned by RNAiFold was required to satisfy IUPAC sequence constraints given by HBVHBGUHVH VHDVBBHDBD BCUGAVGAGV DVBVHBBBVH BHBCGAAACV DBVB as shown in Figure 1 (right); moreover, the MFE structure of each returned sequence, determined by RNAfold 1.8.5, is necessarily identical to the target consensus structure of PLMVd, as shown in Figure 2.

RNAiFold was run four times, each time additionally constraining GC content to be within a specified range. Altogether, over one million solutions of RNA inverse folding were returned before memory exhaustion (using the 32 bit version of run-time system COMET): 200 072 with GC-content 30-39%, 352 924 with GC-content 40-49%, 349 325 with GC-content 50-59%, 366 323 with GC-content 60-69%, constituting a total of 1 268 644 sequences. Output sequences **s** were selected according to a number of criteria explained below.

Measures used in selecting promising hammerhead candidates from RNAiFold were of two basic types that addressed the following questions: (i) To what extent do low energy structures of **s** resemble the MFE structure? (ii) To what extent are the same structural regions of PLMVd AJ005312.1/282-335 as *flexible/rigid* as those of **s**? In other words, the measures used for sequence selection concern either *structural diversity* or regional *structural flexibility/rigidity*; in particular, no sequence homology measures were used in selecting candidate hammerhead sequences for testing, including the program Infernal (60).

One measure of type 1 is the Boltzmann probability *P*(*S*_0_, **s**), where *S*_0_ denotes the MFE structure of **s** (identical to the Rfam consensus structure of PLMVd AJ005312.1/282-335, since RNAiFold solves inverse folding), and $P(S_0,{\bf s}) = \frac{\exp (-E(S_0,{\bf s})/RT}{Z}$, where *E*(*S*_0_, **s**) is the free energy of structure *S*_0_ for sequence **s**, as computed by Turner 1999 energies, and *Z* is the partition function. Other measures of type 1 are average structural positional entropy (61), ensemble defect (62), expected base pair distance (50), Vienna structural diversity (28), Morgan-Higgs structural diversity (63). Additionally, the restriction of these measures to the positions 6-8, 22-25, 27-29, 44-49, was computed. Throughout this paper, we use the term *conserved site* to denote these 16 positions (we use the term *conserved site*, rather than *active site*, which has a different meaning in the biochemical literature). Thus we included measures such as average (structural positional) entropy of conserved site, ensemble defect of conserved site, etc. Measures of type 2 concern the maximum discrepancy between values of type 1 for a candidate sequence **s** and wild-type PLMVd AJ005312.1/282-335. These are briefly explained in the next section; see (50,51) or SI.

#### Structural positional entropy

In selecting the most promising candidate hammerheads from the sequences returned by RNAiFold, we additionally considered *discrepancy* (deviation) from structural positional entropy of conserved positions in PLMVd. Unlike the notion of nucleotide positional entropy used in sequence logos (64), structural positional entropy is defined as follows. If *n* is the length of a given RNA sequence, then for 1 ≤ *i*, *j* ≤ *n*, let $p^*_{i,j}$ denote the probability *p*_*i*, *j*_ of base pair (*i*, *j*) if *i* < *j*, the probability *p*_*j*, *i*_ of base pair (*j*, *i*) if *j* < *i*, and the probability that *i* is unpaired, *i* = *j*. With this notation, the (structural) entropy of position *i* is defined by $H(i) = - \sum _{j} \left( p^*_{i,j} \log p^*_{i,j} + (1-p^*_{i,j}) \log (1-p^*_{i,j}) \right)$. Base 2 logarithms are usually used, whereby entropy is given in bits, ranging from a minimum value of 0, where $p^*_{i,j_0}=1$ for some *j*_0_, to a maximum value of ln *n*/ln 2, in the case that $p^*_{i,j}=1/n$ for each *j*.

An alternative to (full) structural positional entropy is binary structural positional entropy, defined by $H_b(i) = -\left( p^*_{i,i} \log p^*_{i,i} + (1-p^*_{i,i}) \log ( 1-p^*_{i,i})\right)$. Binary positional entropy values *H*_*b*_(*i*) range from a minimum value of 0 bits, where position *i* is either always base paired (though possibly to distinct partners) or always unpaired in the low energy ensemble of structures, to a maximum value of 1, where position *i* is paired (unpaired) with exactly probability 1/2. Figure 2 displays full and binary structural positional entropy for PLMVd AJ005312.1/282-335.

At the 16 conserved positions 6–8, 22–25, 27–29, 44–49 of PLMVd, there is a range of structural positional entropy values, suggesting that certain nucleotides may be located within a more flexible (high entropy) region of the structure, while other nucleotides may be located within a more rigid (low entropy) region. Figure 2 indicates the structural entropy of nucleotides within the consensus structure of PLMVd by appropriate colors, as well as a function of position.

Hypothesizing that low [resp. high] entropy regions of the hammerhead ribozyme could indicate structural *rigidity* [resp. *flexibility*] requirements necessary for hammerhead function, we scrutinized the sequences returned by RNAiFold by measures of *deviation (or discrepancy) from structural positional entropy* of PLMVd AJ005312.1/282-335. This led to a number of measures, formally defined in Section 1 of the Supplementary Information, of which the most important are the following: full/binary entropy discrepancy for complete sequence defined in Supplementary Information equations (7) and (8), full/binary entropy discrepancy for the conserved site defined in Supplementary Information equations (20) and (21) (recall that ‘conserved site’ denotes the 16 positions 6-8, 22-25, 27-29, 44-49 constrained by RNAiFold). *Entropy discrepancy* for the complete sequence [resp. conserved site] is defined to be the maximum, taken over all 54 positions [resp. over positions 6–8, 22–25, 27–29, 44–49], of the absolute value of the difference between structural entropy of a candidate returned by RNAiFold and that of PLMVd.

#### Sequences selected

Table 1 shows the candidate hammerhead sequences finally selected for cleavage assay, together with the selection criteria used for each sequence. Ten candidate hammerheads were selected: HH1–HH10. HH1–HH5 were chosen from sequences of specific GC-content ranges, to have have the smallest *binary* entropy discrepancy for the ‘conserved site’. HH1 was selected from sequences having GC-content 30–39%; HH2 from sequences having GC-content 40–49%; HH4 from sequences having GC-content 50–59%; HH5 from sequences having GC-content 60–69%. Since PLMVd AJ005312.1/282-335 has GC-content of 40.7%, HH3 was chosen to have second smallest *binary* entropy distance for the conserved site, selected from sequences having GC-content 40–49%.

Additional candidate hammerheads were chosen by different criteria, in order to determine their effect on functionality. HH6 was chosen to have the *largest* *binary* entropy discrepancy for the conserved site, selected from all sequences having C at cleavage position 8, provided that the Boltzmann probability of the MFE structure exceeded 40%. HH7 was chosen to have the *smallest* ensemble defect of all sequences having C at cleavage position 8. HH8 was chosen to have the smallest ensemble defect of all sequences, regardless of nucleotide at position 8 (HH8 has A at cleavage site, instead of C). HH9 was chosen to have the smallest *binary* entropy discrepancy for the ‘conserved site’, selected from all sequences, for which the probability *P*(*S*_0_, **s**) of the target PLMVd structure was *at most*0.2. Finally, HH10 was chosen to have the smallest *binary* entropy discrepancy for the conserved site, selected from all sequences, regardless of probability of target structure. Note that HH1–HH6 were selected with the requirement that *P*(*S*_0_, **s**) ≥ 0.4, while HH7–HH10 were selected without this requirement. This was done in order to determine how important target structure probability might be in hammerhead functionality.

#### Computational pipeline summary

The following computational pipeline summarizes the generation and selection of candidate hammerhead sequences.

(i) find Rfam sequence, whose MFE structure resembles family consensus structure

(ii) determine highly conserved positions in reliable multiple alignment

(iii) run RNAiFold to solve the constrained inverse folding problem

(iv) filter using Boltzmann probability, GC-content, entropy, ensemble defect, etc.

(v) perform biochemical validation

A Python program can be downloaded from the RNAiFold web site, that automates steps (i) and (ii). Of course, one can bypass step (i) without using Rfam and instead use any reliable multiple sequence/structure alignment.

#### Design of modular hammerhead within another structure

It has many times been observed that aptamers, hammerheads and other functional RNAs constitute *modules*, capable of function even when engineered to form part of a larger RNA molecule. For instance, Wieland et al. (65) created artificial *aptazymes* by replacing a hammerhead helix by a theophylline aptamer, and Saragliadis et al. (66) created artificial *thermozymes*, created by fusing a theophylline aptamer to a *Salmonella* RNA thermometer (66).

**Figure 3.**
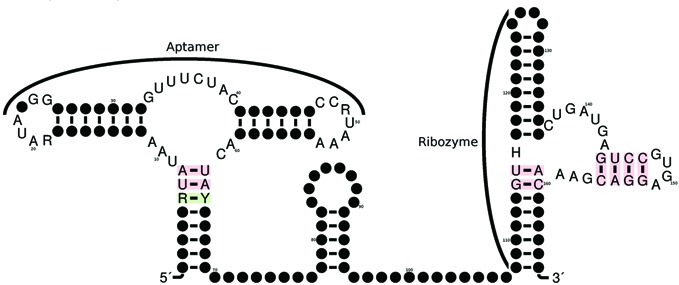
Target secondary structure *S* for modular placement of artificial hammerhead within larger RNA molecule. The structure and highly conserved nucleotides (sequence constraints) of the XPT-riboswitch appear on the left, while the structure and highly conserved nucleotides of the type III hammerhead ribozyme appear on the right.

With the intent of designing a guanine-activated riboswitch with a modular hammerhead, we followed the following steps in rationally designing a synthetic 166 nt RNA, with putative type III hammerhead module. Target secondary structure *S* was taken to be the structure of the gene off xanthine phosphoribosyltransferase (XPT) riboswitch, depicted in Figure 1A of (67), whereby the terminator loop (expression platform) was replaced by the Rfam consensus structure for a type III hammerhead. Sequence constraints were chosen to be the highly conserved nucleotides of the Rfam consensus structures for the purine riboswitch (RF00167 seqcons view of consensus structure) and for type III hammerhead (RF00008 seqcons view of consensus structure). Figure 3 displays the target structure *S* for computational design of a modular hammerhead within the terminal stem-loop of a structure similar to the XPT riboswitch. We gave RNAiFold an additional compatibility constraint, whereby returned sequences were required to be compatible to a second structure *S*′, in which the hammerhead cleavage site (NUH) is fully sequestered within a base-paired region. Positions 60–118 of *S*′ are given as follows:

**Figure:**
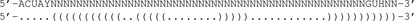


while all positions in *S*′ outside of 60–118 (i.e. from 1–59 and 119–166) are unpaired.

We filtered sequences output by RNAiFold, by applying RNAbor (68), and its faster sequel, FFTbor (69). Given reference structure *S*, RNAbor and FFTbor return the *density of states* with respect to *S*, which depicts the Boltzmann probability $p(k)=\frac{Z_k}{Z}$ for secondary structures to have base pair distance *k* from *S*. Additionally, RNAbor computes, for each *k*, the MFE_*k*_-structure; i.e. that structure having MFE over all structures whose base pair distance from the reference structure *S* is exactly *k*.

From a partial output of 3000 sequences from RNAiFold, only one sequence **s** satisfied the following two properties, when applying RNAbor with input **s** and reference MFE structure *S*: (i) The density of states figure has a pronounced peak at *k* = 0, corresponding to the location of the MFE structure *S*; (ii) There was another pronounced peak for value *k* ≫ 0, corresponding to a structure *T* containing the base pairs in *S*′, which thus should sequester the ribozyme cleavage site NUH, located at position 114–116—see Supplementary Information Figure S3.

The final, selected sequence 166 nt **s** is given as follows: GCCGC GUAUA AGGGC UGCGA UAAGG GCAGU CCGUU UCUAC GGGCG GCCGU AAACC GCCCA CUACG CGGCG UGGUU AAGCC GGAAA GGAGA CCGGC AGGAG GGUAA UGGGC CGCGU CGCGG CGCGG GAGCG CGCCG CCUGA UGAGU CCGUG AGGAC GAAAC GCGGCC.

### Experimental validation

Complementary DNA oligonucleotides, corresponding to the DNA sequence of the designed RNAs preceded by a T7 RNA polymerase promoter, were purchased from MWG Operon. The 10 hammerhead candidate sequences HH1–HH10, extended 2 nt on the left by GG and 2 nt on the right by CC for transcriptional efficiency, and the 166 nt sequence, harboring a candidate hammerhead in the rightmost stem-loop of Supplementary Information Figure S3 were constructed using primer extension and PCR amplified (5 U taq polymerase (New England Biolabs), 2.5 mM each NTP, 1x NEB Thermopol buffer). For each of the 10 designed hammerhead sequences, the H8G mutant was constructed in a similar manner, using alternative oligonucleotides containing the mutation. Similarly, C116G (analogous to H8G) and G142U mutations were constructed for the 166 nt designed ribozyme. The resulting PCR products were TOPO-cloned (Invitrogen), and the designed and mutant sequences were verified by sequencing plasmids containing full-length PCR products. These plasmids were subsequently used as templates for PCR reactions to generate template for *in vitro* transcription.

To generate the RNA, *in vitro* transcription was performed using T7 RNA polymerase (400 U T7 polymerase, 80 mM HEPES-KOH pH 7.5, 24 mM MgCl_2_, 2 mM spermidine, 40 mM DTT, 2 mM each NTP) with the addition of 10 μCi of α-32P-GTP for transcriptions to generate body-labeled RNA when necessary. To prevent premature cleavage during transcription, 100 uM of oligonucleotides complementary to nucleotides 17–35 (numbering starts after the leading GG) were added to each reaction. Full-length RNAs were purified using denaturing polyacrylamide gelelectrophoresis (PAGE) (20% acrylamide).

To assess self-cleavage of designed hammerhead sequences, RNA was incubated for 1 h in cleavage buffer (5 mM MgCl_2_, 50 mM tris pH 7.5) at 25°C. Subsequently, 1 volume of 2x gel-loading buffer (16 M urea (supersaturated), 10 mM ethylenediaminetetraacetic acid (EDTA), 20% sucrose, 0.1% sodium dodecyl sulphate (SDS), 100 mM tris pH 8.0, 100 mM borate, 0.05% bromophenol blue) was added to quench the reaction with final urea and EDTA concentrations of 8 M and 5 mM respectively. The reaction was placed on ice until gel loading.

Samples lacking Mg^++^ were incubated in 50 mM tris pH 7.5 for 1 h at 25°C. For the 166 nt RNA, cleavage experiments were conducted under similar conditions but reactions were incubated for a few seconds (0 h), 30 min, 5 h and 24 h, and samples lacking Mg^++^ were incubated in 50 mM tris pH 7.5 for 24 h at 25°C. Cleavage products were separated by denaturing PAGE (10% acrylamide), and the gels dried prior to exposure to phosphoimager plates (GE Healthcare) for 18 h. The gels were imaged using a STORM 820 phospoimager (GE Healthcare).

#### Kinetics

To determine the cleavage rates for designed hammerhead sequences, body-labeled RNA was incubated in cleavage assays as described above for varying amounts of time. Cleavage products were separated and gels imaged as described above. The cleavage products were quantified using ImageQuant software (GE Healthcare). To calculate the fraction cleaved at time *t*, *F*(*t*), the sum of the quantified counts for 5′ and 3′ cleavage product bands was divided by the total quantified counts for the entire reaction (uncleaved, 5′ and 3′ cleavage products).

The observed cleavage rate *K*_obs_ was computed by using the Matlab function nlinfit with constant error model to fit cleavage time series data using the equation

(1) \begin{equation*} F_{\mbox{}\max }-F(t) = (F_{\mbox{}\max }-F(0)) \cdot \exp ( K_{\mbox{}{\rm obs}}\cdot t) \end{equation*}

where *F*(*t*) denotes the amount of cleavage product measured at time *t*, and *F*_max _ the maximal fraction cleaved. The 95% confidence interval of this fit was calculated from the resulting residuals and variance-covariance matrix using the Matlab function nlpredci. See Supplementary Information Figure S2.

### RESULTS

**Figure 4.**
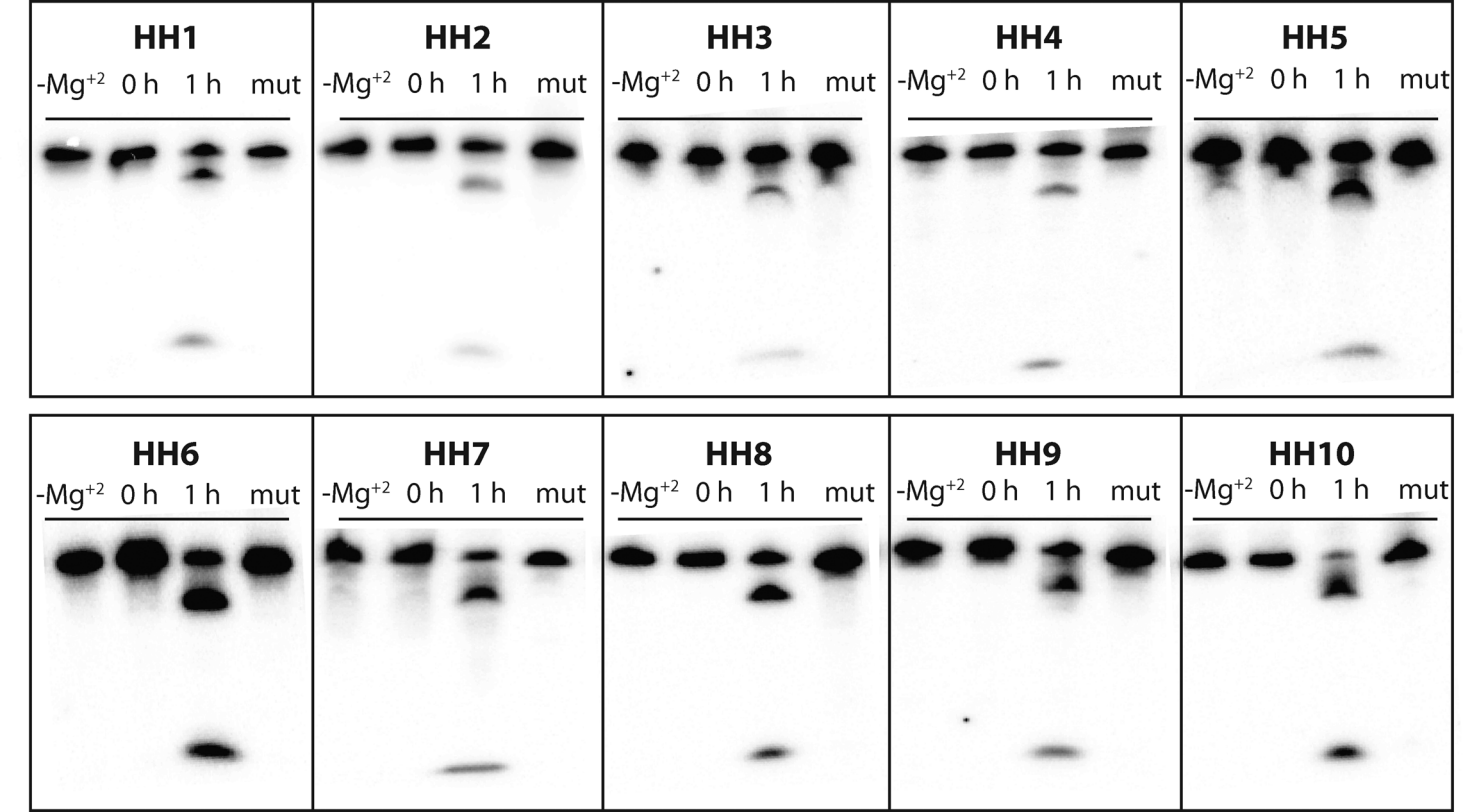
Summary of designed hammerhead cleavage. Each designed hammerhead RNA was incubated under mild conditions for 1 h as described in the ‘Materials and Methods’ section to assess cleavage. As negative controls, a no magnesium and a 0-h reaction were also conducted for each RNA. Additionally, the 8G mutation, predicted to be incompatible with the hammerhead structure (see ‘Materials and Methods’ section), was constructed for each designed sequence and examined under equivalent conditions to confirm that self-cleavage occurs using the expected hammerhead mechanism.

**Figure 5.**
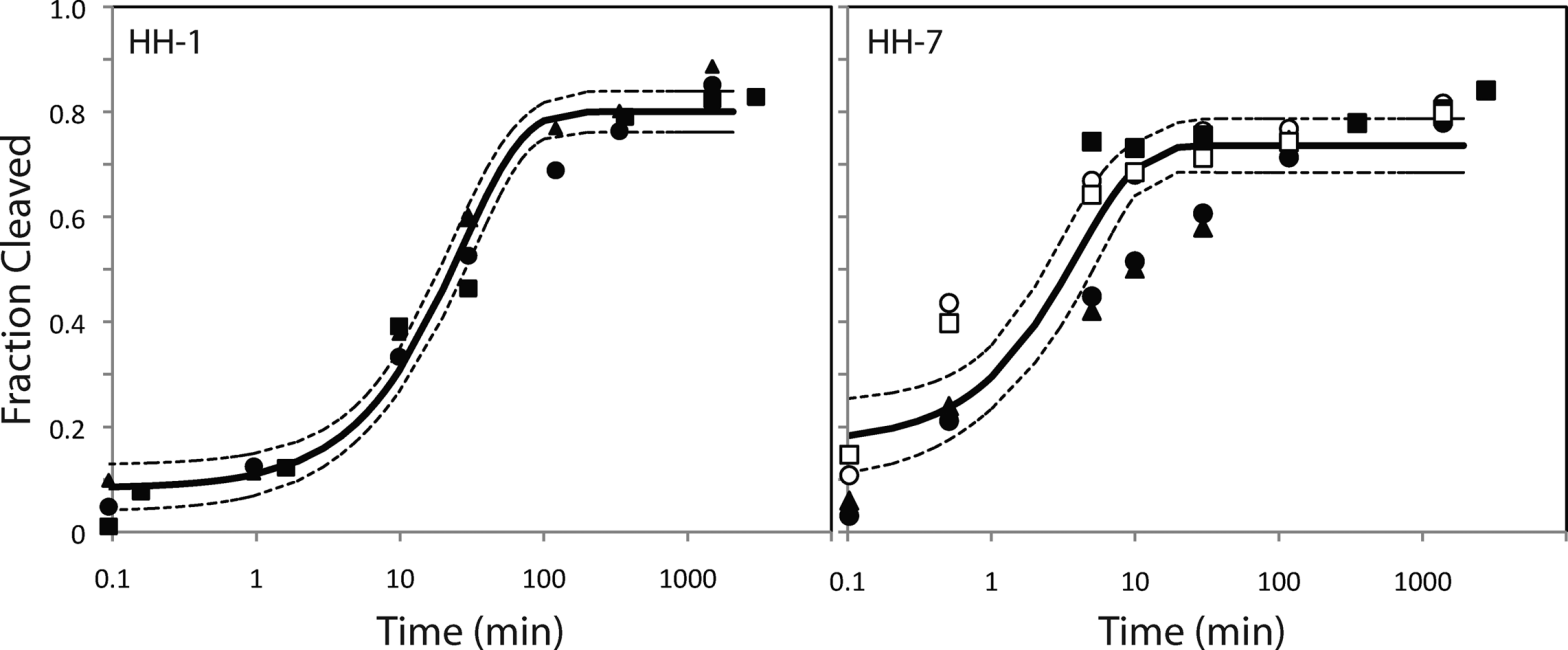
(Left) HH1: typical cleavage time series curve with good error parameters (standard deviation <10% of mean, with mean squared error (MSE) = 0.0029). Solid line represents fitted line, and dotted lines indicate 95% confidence interval. Different datasets represented by filled and unfilled squares, triangles, etc. (Right) HH7: fastest hammerhead cleavage rate, though determined with considerable error (MSE = 0.01). In data from the first experiments for HH7, indicated by filled squares, cleavage had been measured at times when maximum cleavage had nearly occurred (these points appear in the flat part of the fitted curve). Subsequent datasets have focused on shorter time periods. This curve was fitted using five datasets. Time series curves for cleavage data for the remaining eight designed hammerheads HH2-HH6 and HH8-HH10 are shown in Supplementary Information.

Given the target Rfam consensus structure *S* of PLMVd AJ005312.1/282-335, which is identical with the MFE secondary structure using RNAfold 1.8.5, 16 highly conserved positions nucleotides were taken as constraints in the generation of over 1 million sequences solving the inverse folding problem, as determined by RNAiFold 1.8.5. Using distance measures of *dissimilarity* of low energy structures to the MFE structure (positional entropy, ensemble defect, structural diversity, etc.) together with measures of molecular structural flexibility/rigidity, 10 putative hammerhead sequences were selected for *in vitro* validation using a cleavage assay. The selected sequences and selection criteria are given in Table 1. All 10 hammerhead candidates, listed in this table, were shown to be functional, with cleavage rates listed in Table 2. Cleavage assay gel images for the designed hammerheads HH1–HH10 are displayed in Figure 4, where each sequence shows Mg^++^-dependent cleavage. In addition, the H8G mutant of each designed hammerhead shows no activity. These data strongly suggest that the designed sequences HH1–HH10 behave in a manner consistent with the expected mechanism for hammerhead ribozymes. Time series for cleavage fraction and kinetics curves for a typical designed hammerhead ribozyme (HH1) and the fastest designed ribozyme (HH7) are shown in Figure 5, while similar figures for the remaining designed hammerheads appear in Supplementary Information Figure S2. Kinetics for the designed hammerheads should be compared with wild-type hammerhead kinetics, where under standard conditions of 10 mM MgCl_2_, pH 7.5 and 25°C, cleavage rates between 0.5 and 2 per minute have been observed for at least 20 different hammerheads (70). It follows that kinetics of the computationally designed hammerheads described in this paper are slower than wild-type hammerheads approximately by a factor of 10.

**Table 2.** Kinetics of cleavage for 10 computationally designed hammerheads and correlation with several measures

ID	*K*_obs_	*F*_max_	MSE	Pos ent	Ens def	EBPD dis act
HH1	0.037	0.79	0.0029	0.270882	4.167687	0.0501207
HH2	0.0057	0.74	0.003	0.287235	4.552053	0.0386253
HH3	0.0027	0.65	0.0039	0.259577	4.121914	0.0410984
HH4	0.0127	0.55	0.0048	0.403846	6.755976	0.0354213
HH5	0.0085	0.52	0.0066	0.382235	6.240083	0.033132
HH6	0.102	0.73	0.0047	0.414872	8.138131	0.059864
HH7	0.25	0.74	0.0107	0.119159	2.383671	0.0406728
HH8	0.02	0.68	0.0124	0.078518	1.45179	0.0662421
HH9	0.025	0.76	0.0015	0.247886	4.525597	0.0328018
HH10	0.14	0.77	0.01	0.286425	4.975979	0.0269354

Cleavage rate *K*_ob__s_ (min^−1^), maximum percent cleavage *F*_max_ , mean squared error MSE, (full) structural positional entropy Pos Ent, ensemble defect Ens Def and expected base pair distance discrepancy for the ‘conserved (or active) site’ EBPD Dis Act. The Pearson correlation between cleavage rate and Pos Ent, Ens Def, EBPD Disc Active is respectively −0.461, −0.370, −0.438; i.e. cleavage rate is faster when these secondary structure deviation values are smaller. Other measures, such as structural diversity, had smaller correlation, while measures such as GC-content and MFE had almost no correlation with cleavage rate. See Supplementary Information for full table of correlation for all measures.

Pearson correlation coefficient was determined between cleavage rate *K*_*obs*_, obtained by fitting equation (1) with data from three to five technical relicates, and 21 measures, including average positional entropy, GC-content, MFE, etc. See Supplementary Information for all correlation values. The most pronounced correlations were observed between *K*_*obs*_ and (full) average structural positional entropy, ensemble defect, and expected base pair distance discrepancy for ‘conserved site’ with values respectively of −0.461, −0.370, −0.438; i.e. cleavage is faster when these measures are smaller. See Supplementary Information equations (7), (5) and (22) for formal definitions of these notions.

**Figure 6.**
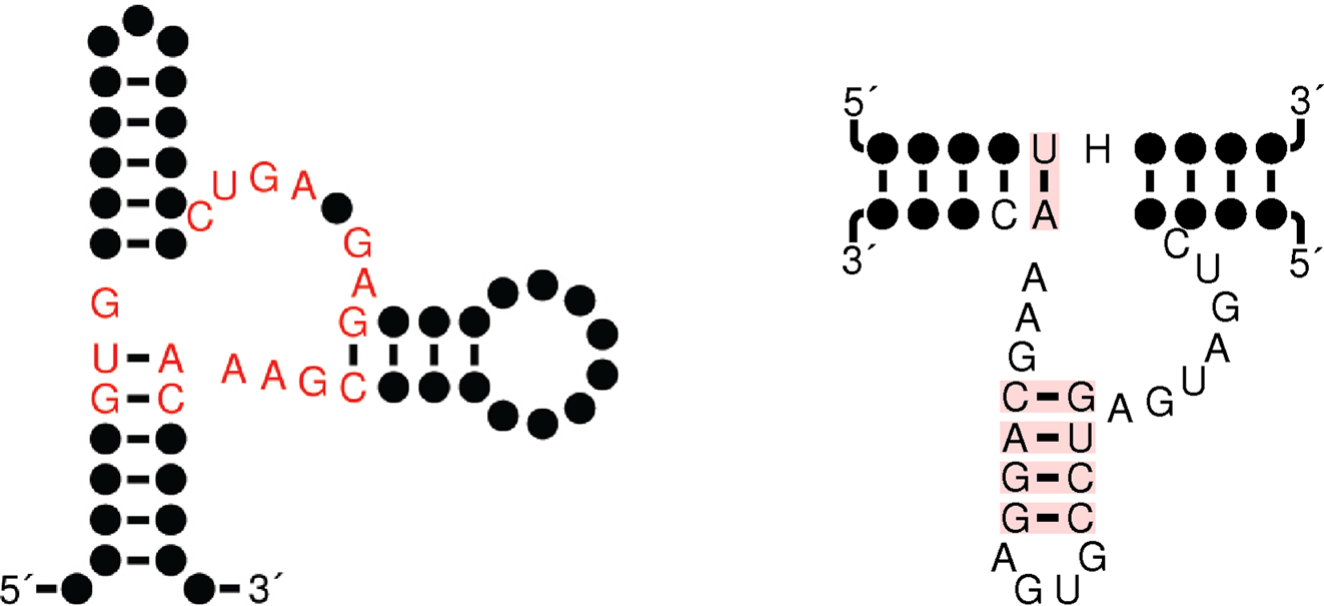
(Left) Target structure *S* used in computational experiment with RNAiFold, which determined that *no sequence exists*, having guanine at the cleavage site 8 along with those 15 nucleotides of Peach Latent Mosaic Viroid (PLMVd) AJ005312.1/282-335 having sequence conservation exceeding 96%, and which the Rfam consensus structure of PLMVd (i.e. whose RNAfold 1.8.5 MFE structure is the consensus secondary structure of of PLMVd). (Right) Hammerhead ribozyme (lower molecule) hybridized with *trans*-cleavage target RNA (upper molecule). Cleavage site NUH occurs at position 4–6 of the upper molecule, where ‘H’ denotes ‘not G’. RNAiFold shows that no two sequences **s**_1_, **s**_2_ exist, where **s**_1_ contains ‘GUG’ at positions 4-6, both **s**_1_, **s**_2_ contain the other indicated nucleotides, for which the indicated structure is the MFE hybridization of **s**_1_, **s**_2_. The nonexistence, as determined by RNAiFold, of any sequence folding into target structure *S*, which has GUG at the cleavage site and satisfies certain additional minimal constraints, strongly suggests that GUG is not a hammerhead cleavage site is due to the inability of the molecule to fold into a structure necessary for nucleophilic attack. Image of right panel adapted from figure 3A from (72), and both images produced by R2R (75).

It is known from literature (58,59) that hammerhead cleavage sites are of the form NUH (e.g. GUH and CUH, but not GUG). Indeed, Carbonell et al. (71) suggest that G8 would pair with C22 (in our numbering) and impede its role in the catalytic pocket. Figure 4 shows that the H8G mutant of each designed sequence HH1–HH10 does not cleave under mild denaturing conditions that suffice for cleavage of HH1–HH10. In addition, RNAiFold determined that (provably) there is no RNA sequence, whose MFE structure is the Rfam consensus structure of PLMVd AJ005312.1/282-335, having a guanine at cleavage site 8, as well as the 15 highly conserved nucleotides of PLMVd at positions 6–7, 22–25,27–29, 44–49 (left panel of Figure 6). This result holds for both the Turner 99 and Turner 2004 energy models.

Since RNAiFold also solves the inverse hybridization problem, we considered the NUH cleavage target of *trans*-cleaving hammerhead ribozymes, known from comparative sequence analysis (72). Application of RNAiFold showed that there do not exist any two sequences, where the first contains GUG at the cleavage site location, for which the MFE hybridization structure is the target structure appearing in the right panel of Figure 6. Taken together, these results provide a compelling computational explanation for the reason that GUG is not a hammerhead cleavage site.

To demonstrate the functionality of a computationally designed hammerhead, occurring within a larger rationally designed RNA, we synthesized the 166 nt sequence **s**, designated as ‘synthetic wild-type’, as well as two mutant sequences **s**_1_, **s**_2_, each containing a mutation that should inactivate hammerhead activity. Sequence **s**_1_ contains a C116G mutation at the GUC site of cleavage, while **s**_2_ contains a G142U mutation in a distal section of the ribozyme, known to be required for cleavage (the CUGAUGA sequence). Cleavage assays under mild conditions (5 mM MgCl_2_, 50 mM tris pH 7.5, 25°C) show that ∼40% of our synthetic wild-type sequence rapidly cleaves at the expected site (see Supplementary Information Figure S4 for T1 mapping of the cleavage products), in the absence and presence of guanine.

**Figure 7.**
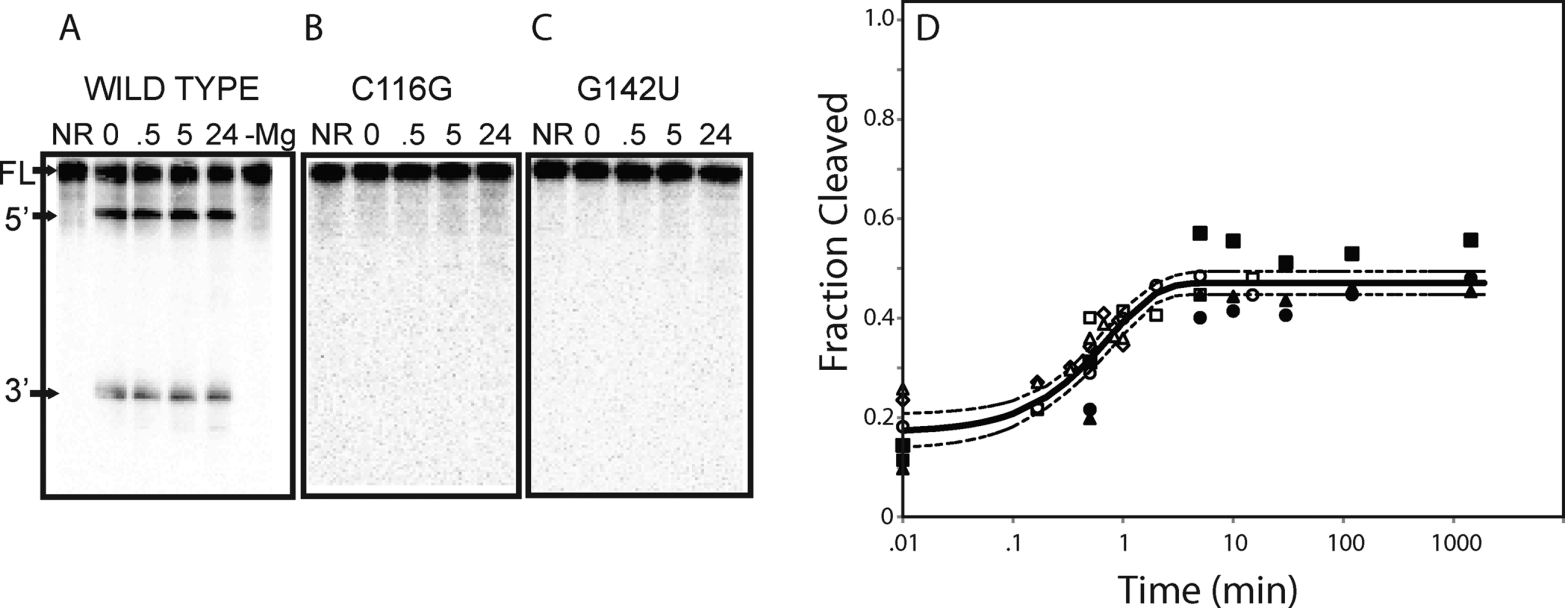
(Left) Cleavage assay reactions (**A**,**B**,**C**) of designed hammerhead (wild-type), mutant C116G and mutant G142U. For the wild-type (A), mutant C116G (B), and mutant G142U (C) gel images, lane 1 is the undigested RNA (full-length, FT), lanes 2–5 are reactions in cleavage buffer (50 mM Tris pH 7.5, 5 mM MgCl_2_) at the 0 s, 30 min, 5 h and 24 h time points respectively (5′ and 3′ cleavage products indicated). For the wild-type (A), lane 6 is a reaction lacking Mg (50 mM tris pH 7.5) incubated for 24 h. It is evident that cleavage only occurs for the wild-type sequence, and when Mg is present. (Right) Cleavage time series curve (D) for the 166 nt designed hammerhead, with observed cleavage rate of 1.3/min with an *F*_max_ of 0.47 and MSE of 0.0026. This construct displays kinetics comparable with that of wild-type hammerheads, although the cleavage amount *F*_max_ is much lower than that of wild-type hammerheads.

The cleavage is Mg^++^-dependent (Figure 7A), and the hammerhead appears to cleave rapidly within seconds. Neither of the mutant sequences displays any cleavage under the same conditions, even with significantly longer incubation times (Figure 7B,C). Kinetics for the 166 nt synthetic ribozyme are comparable with those of wild-type hammerheads, with an observed cleavage rate *K*_obs_ of 1.3/min and *F*_max_ of 0.47 (Figure 7D). Addition of 1 mM guanine has no significant affect on either the *K*_obs_ or the *F*_max_ (Supplementary Information Figure S4); i.e. the designed riboswitch was constitutively on.

## DISCUSSION

In this paper, we have demonstrated the success of a purely computational approach for the rational design of artificial type III hammerhead ribozymes. Figure 4 clearly shows the Mg^++^-dependent cleavage of each designed sequence HH1-HH10, as well as the non-cleavage of the 8G mutant of each sequence, strongly suggesting that cleavage is due to the usual hammerhead mechanism. Cleavage time series data for three to five technical replicates for each of the 10 computationally designed hammerheads, displayed in Figure 5 and Supplementary Information Figure S2, lead to observed cleavage rates varying 100-fold from 0.0027 min^−1^ for HH3, to 0.25 min^−1^ for HH7. The relatively fast cleavage rate of HH7, selected from over 1 million sequences returned by RNAiFold solely on the criteria of minimizing ensemble defect, *with* the additional requirement of having GUC at the cleavage site, is slower only by a factor of 10 from wild-type hammerhead cleavage rates (recall that wild-type cleavage rates vary between 0.5 and 2 per minute (70)). In contrast, HH8 had an observed cleavage rate of 0.02 min^−1^, although it was selected solely on the criteria of minimizing ensemble defect—*without* the additional requirement of having GUC at the cleavage site. This experimental result suggests that cleavage kinetics may be the underlying reason that cytidine is present at cleavage position 8 in 95% of the 84 sequences in the Rfam seed alignment of family RF00008.

Among more than 20 computational features, the features found to be most highly correlated with cleavage rate *K*_*obs*_ for HH1-HH10 were (full) average structural positional entropy, ensemble defect and expected base pair distance discrepancy for ‘conserved site’ with values respectively of −0.461, −0.370, −0.438. However, this result is based on a tiny set of data and can only be taken as a suggestive first step toward a more systematic determination of which measures of structural diversity/flexibility/rigidity might best predict ribozyme activity.

In the design phase, we selected HH1-HH5 to have a positional entropy profile similar to that of wild-type PLMVd, i.e. to have small average (structural positional) entropy of conserved site, based on the intuition that certain positions in the wild-type hammerhead may have high entropy to support cleavage. However, it is presently unclear whether discrepancy measures (absolute difference between wild-type and synthetic) restricted to the conserved site are useful at all. Indeed, among all sequences returned by RNAiFold, HH6 had an observed cleavage rate of 0.102/min, a bit less than half that of HH7, yet HH6 was selected to have the *largest* entropy discrepancy from the conserved site among all sequences, such that the probability of the MFE structure exceeded 40%. Without additional experiments on a large collection of computationally designed hammerheads, and perhaps without extensive molecular dynamics modeling, it remains unclear to what extent hammerhead efficiency, as assayed by cleavage kinetics, is dependent on matching the positional stability and flexibility of the wild-type PLMVd hammerhead.

It is interesting to note that HH1-HH6 are not recognized as hammerheads by the Rfam web server (54), which relies on the program Infernal (60), a sophisticated machine learning algorithm (stochastic context free grammar) that depends on recurring sequence and structural motifs. Rfam predicts only HH7-HH10 to be type III hammerheads, with the following confidence scores: HH7 41.3 bits (*E*-value 5.9e-09), HH8 38.1 bits (*E*-value 4.6e-08), HH9 37.5 bits (*E*-value 6.8e-08), HH10 38.9 bits (*E*-value 2.9e-08).

Currently, NUPACK-DESIGN (47) appears to be one of the most efficient tools to design RNAs by employing a heuristic computational search to minimize ensemble defect. Given the constraints for synthetic hammerhead design described in this paper, the NUPACK server returned 10 sequences, nine of whose MFE structures were identical to that of PLMVd AJ005312.1/282-335. (The NUPACK philosophy is that minimizing ensemble defect is more important than guaranteeing that sequences be an exact solution of the inverse folding problem. The NUPACK web server has an upper limit of 10 sequences that can be returned. In contrast, after downloading and compiling the NUPACK source code, each run of NUPACK design returns a single sequence; since the procedure is stochastic, repeated runs will usually return different sequences.) The first sequence returned by the NUPACK web server was CGCCGGUAGC CUGACCCAGG CCUGAAGAGC UCUACCCCCC GAGCGAAACC GGCU, which has normalized ensemble defect of 2.5%, the same value as that of HH8 (1.45179/54 = 0.025030862). The cleavage rate of HH8, whose cleavage site is GUA (as in the NUPACK sequence) is 0.02/min, with five faster cleaving synthetic hammerheads. Despite the speed of NUPACK in designing RNAs with low ensemble defect, one advantage of RNAiFold is that prioritization of candidate sequences is performed in a postprocessing phase, thus allowing one to select solutions of inverse folding that are optimal with respect to various measures (not only ensemble defect), as we have done in this paper.

We have additionally tested the programs RNAdesign (73) and IncaRNAtion (74), with the Rfam consensus structure of PLMVd hammerhead as target structure. Only 5.84% [resp. 2.57%] of the sequences returned by RNAdesign using eos(1) [resp. IncaRNAtion] actually folded into the target structure, thus requiring substantial additional computation time to select those sequences that fold into the target (in constrast, RNAiFold returns only sequences that correctly fold into the target structure). See Supplementary Information and http://bioinformatics.bc.edu/clotelab/SyntheticHammerheads/ for comparative results concerning entropy, ensemble defect, etc.

In addition to computationally designing the functional hammerheads HH1-HH10, we have designed the 166 nt sequence **s**, in which a synthetic hammerhead is embedded within the terminal stem-loop of the structure depicted in Figure 4. The sequence **s** is self-cleaving at the expected GUC cleavage site 114–116. Moreover, as shown in Figure 7D, cleavage kinetics for this 166 nt artificial ribozyme (*K*_obs_ = 1.3/min) are as fast as those of wild-type hammerheads, although the cleavage amount (*F*_max_ = 0.47) is quite poor compared with our other designed ribozymes HH1–HH10. By utilizing two mutants, one at the cleavage site position 116, and one further downstream at position 142 in the CUGUAGA segment necessary for catalysis of cleavage, we show effectively that cleavage in the synthetic wild-type, designed construct is due to the usual hammerhead mechanism. Additionally, we have demonstrated Mg^++^-dependence, necessary for the cleavage mechanism, through the complete absence of 5′- and 3′-cleavage products when incubated for an extended period of time of 24 h in buffer lacking Mg^++^.

The software RNAiFold solves the inverse folding problem, not only for a target secondary structure, but as well when the target *S* is the hybridization of two secondary structures; i.e. when *S* contains both intra- and inter-molecular base pairs. Since RNAiFold uses constraint programming, it can perform a complete search of the space of compatible sequences, and thus return *all* sequences, whose MFE structure [resp. MFE hybridization] is a given target structure [resp. hybridization], or *can certify that no such solution exists*. The fact that RNAiFold determined that no solution of inverse folding exists for the GUH to GUG [resp. NUH to GUG] mutant of the target structure depicted in Figure 3 [resp. the right panel of Figure 6] provides very compelling computational evidence that there are structural reasons for the reason that GUG is not a hammerhead cleavage site.

## CONCLUSION

In this paper, by employing our constraint programming solution RNAiFold (50,51) to generate >1 million sequences, that agree with PLMVd AJ005312.1/282-335 at the 15 nucleotides having >96% conservation in Rfam RF00008 seed alignment, and have MFE structure identical to that of the Rfam consensus secondary structure of PLMVd. Ten candidate hammerheads, which were selected using criteria that measure either *structural diversity* or regional *structural flexibility/rigidity*, were shown to be functional, with varying kinetics, by an *in vitro* cleavage assay. This appears to be the first purely computational design and experimental validation of novel functional ribozymes. Moreover, by computationally designing a 166 nt synthetic RNA, whose terminal stem-loop harbors a functional computationally designed hammerhead, we show that *in silico* design and placement of artificial hammerheads is possible.

Since RNAiFold supports user-defined sequence constraints, as well as structural compatibility and incompatibility constraints, our method should be able to rationally design hammerheads that reside within larger RNAs, which meet user-defined sequence and structure constraints.

## SUPPLEMENTARY DATA

Supplementary Data are available at NAR Online.

SUPPLEMENTARY DATA
