# Supplementary material for: Complete RNA inverse folding: computational design of functional hammerhead ribozymes
Source: Nucleic Acids Res. 2014 Sep 10;42(18):11752–62. doi: 10.1093/nar/gku740 (PMC4191386; doi:10.1093/nar/gku740)
Supplement: SUPPLEMENTARY DATA [file supp_42_18_11752__index.html]

Complete RNA inverse folding: computational design of functional hammerhead ribozymes — SUPPLEMENTARY DATA 

# Complete RNA inverse folding: computational design of functional hammerhead ribozymes

## SUPPLEMENTARY DATA

**Files in this Data Supplement:**

- SUPPLEMENTARY DATA
