# Supplementary material for: Complete RNA inverse folding: computational design of functional hammerhead ribozymes
Source: Nucleic Acids Res. 2014 Sep 10;42(18):11752–62. doi: 10.1093/nar/gku740 (PMC4191386; doi:10.1093/nar/gku740)
Supplement: SUPPLEMENTARY DATA [file supp_gku740_nar-00799-r-2014-File004.pdf]

Supplementary Information for "Complete RNA inverse folding: computational design of functional hammerhead ribozymes"

I. Dotu [1], J.A. Garcia-Martin [1], B.L. Slinger [1], V. Mechery [2], M.M. Meyer [1], P. Clote [1] *

[1]Biology Department, Boston College, 140 Commonwealth Avenue, Chestnut Hill, MA 02467 (USA) and [2]Hofstra North Shore-LIJ School of Medicine, Hempstead, NY 11549 (USA).

1 Selection of PLMVd: consensus structure and `RNAfold` 1.8.5

Figure 1 displays the minimum free energy (MFE) structure of type III hammerhead from Peach Latent Mosaic Viroid (PLMVd) AJ005312.1/282-335 (isolate LS35, variant ls16b), taken from Rfam [4] family RF00008. The left [resp. right] panel is the MFE structure computed by Vienna RNA Package `RNAfold` 1.8.5 [resp. 2.0.7]. The 54 nt structure in the left panel closely resembles the 56 nt family consensus structure illustrated at `http://rfam.sanger.ac.uk/family/RF00008#tabview=tab3`.

For an RNA sequence **s** in the Rfam seed alignment of an Rfam family, such as RF00008, we take the *consensus* structure of **s** to be that structure, obtained in the following manner:

1. Place a left [resp right] parenthesis `(` [resp. `)`] in a position in which the Rfam Stockholm format file has a left angle bracket $<$ [resp. $>$] in the same column. All other positions should contain a dot $\bullet$.
2. Remove a left,right parenthesis pair at position i, j if $j - i \leq 3$.
3. Remove a left,right parenthesis pair at position i, j if the nucleotides in positions i, j of **s** do not constitute a Watson-Crick or wobble pair.

Using these steps, we wrote a simple script, `parseRfam.py`, to produce such consensus structures for any Rfam sequence (script available upon request).

The MFE structure of Peach Latent Mosaic Viroid (PLMVd) AJ005312.1/282-335 is identical to its Rfam consensus structure, as just defined, provided that `RNAfold` 1.8.5 is used; the right panel of Figure 1 shows that the MFE structure produced by `RNAfold` 2.0.7 is radically different than the Rfam consensus structure. For this reason, throughout this paper, we have used Turner 1999 energies (`RNAfold` 1.8.5), rather than Turner 2004 energies (`RNAfold` 2.0.7).

Additionally, PLMVd AJ005312.1/282-335 is the only one of the 84 sequences in the seed alignment of RF00008, whose MFE structure is identical to its Rfam consensus structure. Since we wished to apply `RNAiFold` to solve the inverse folding problem for a biologically functional type III hammerhead structure, we selected the secondary structure of PLMVd AJ005312.1/282-335 as target structure. If the MFE structure of every sequence in the seed alignment of RF00008 had been distinct from the Rfam consensus structure, then we would have selected that MFE structure most closely resembling the Rfam consensus structure.

2 Definition of computational measures

Here we define the computational measures appearing in the figures displaying the Pearson correlation with cleavage rates for HH1-HH10 data. Most of these measures depend only on the computation of the base

*To whom correspondence should be addressed. Tel: +1 617 552 1332; Fax: +1 617 552 2011; Email: clote@bc.edu

pairing probabilities

$$p_{i,j} = \sum_{\{S:(i,j)\in S\}} P(S) = \frac{\sum_{\{S:(i,j)\in S\}} \exp(-E(S)/RT)}{Z} \tag{1}$$

where $P(S)$ is the Boltzmann probability of structure S of a given RNA sequence $\mathbf{s} = \mathbf{s}_1, \ldots, \mathbf{s}_n$, $E(S)$ is the Turner 1999 energy of secondary structure S [8, 16], $R \approx 0.001987$ kcal mol^{-1} K^{-1} is the universal gas constant, T is absolute temperature, and the *partition function* $Z = \sum_S \exp(-E(S)/RT)$, where the sum is taken over all secondary structures S of a. The base pairing probabilities $p_{i,j}$ for base pair (i,j), for $1 \leq i < j \leq n$, are computed by McCaskill's algorithm [9] using `RNAfold` 1.8.5 with flag -p. In this paper, we did not use the newer free energy parameters of `RNAfold 2.0.7`, since the MFE structure for PLMVd AJ005312.1/282-335 is radically different, as shown in Figure 1.

For each fixed position $1 \leq i \leq n$, we define the probability distribution $p^*_{i,j}$, for j varying in $[1,n]$, by symmetrizing p for values $1 \leq i,j \leq n$, and then defining $p^*_{i,i} = 1 - \sum_{j>i} p_{i,j} - \sum_{j<i} p_{j,i}$ Finally, for a given reference structure S_0 and $1 \leq i,j \leq n$, define $bp(S_0,i) = j$ if either $(i,j) \in S_0$ or $(j,i) \in S_0$.

Rate(KOBS): Observed cleavage rate, K_{obs}, obtained by fitting data to equation

$$\frac{F_{\max} - F(t)}{F_{\max} - F(0)} = \exp(K_{obs} \cdot t) \tag{2}$$

FMAX: Maximum cleavage proportion, measured after 48 hours, and obtained by using the `Matlab` function `nlinfit` with constant error model to fit cleavage time series data using the equation (2).

ln(Rate(KOBS)): Natural log of K_{obs}.

GCcont: Number of G plus C divided by sequence length.

Energy: Free energy of the MFE structure of sequence, determined by `RNAfold` 1.8.5. The MFE structure is identical to the Rfam consensus structure of PLMVd in present case, since sequences were returned by `RNAiFold`.

ExpBPDist: Expected base pair distance for $\mathbf{s}$ to its MFE structure S_0, defined by

$$EBPD(\mathbf{s}) = \sum_{1\leq i\leq n} EBPD(\mathbf{s}, i) \tag{3}$$

where

$$EBPD(\mathbf{s}, i) = \sum_{1\leq j\leq n, i\neq j} I[bp(S_0,i) \neq j] \cdot p_{i,j} + I[bp(S_0,i) = j] \cdot (1 - p_{i,j}) \tag{4}$$

where I denotes the indicator function. The positional measure $EBPD(\mathbf{s}, i)$ was defined to allow its restriction to nucleotides in an 'conserved site'. Note that the value of $EBPD(\mathbf{s})$ is twice the value of expected base pair distance to S_0, as defined in [3].

EnsDef: Ensemble defect [2] is the expected number of nucleotides whose base pairing status differs from the MFE structure S_0, defined by

$$EnsDef(\mathbf{s}) = n - \sum_{1\leq i,j\leq n} p^*_{i,j} \cdot I[bp(S_0,i) = j] - \sum_{1\leq i\leq n} p^*_{i,i} \cdot I[i \text{ unpaired in } S_0] \tag{5}$$

where I is the indicator function. Define the positional ensemble defect

$$EnsDef(\mathbf{s}, i) = 1 - \sum_{1\leq j\leq n} p^*_{i,j} \cdot I[bp(S_0,i) = j] - \sum_{1\leq i\leq n} p^*_{i,i} \cdot I[i \text{ unpaired in } S_0] \tag{6}$$

so it follows that $EnsDef(\mathbf{s}) = \sum_{i=1}^{n} EnsDef(\mathbf{s}, i)$.

(Full) Entropy: Full average structural positional entropy, defined by

$$\langle H(\mathbf{s})\rangle = \sum_{i=1}^{n} \frac{H(\mathbf{s},i)}{n} \tag{7}$$

where the full structural positional entropy is defined by $H(\mathbf{s},i) = -\sum_{j=1}^{n} p^*_{i,j} \cdot \ln p^*_{i,j}$. Note that $0 \cdot \ln 0$ is taken to be 0.

(Binary) Entropy: Binary average structural positional entropy, defined by

$$\langle H_b(\mathbf{s})\rangle = \sum_{i=1}^{n} \frac{H_b(\mathbf{s},i)}{n} \tag{8}$$

where the binary structural positional entropy at position i is defined by $H_b(\mathbf{s},i) = -\left(q_i \cdot \ln q_i + (1-q_i) \cdot \ln(1-q_i)\right)$. Here, $q_i = p^*_{i,i} = 1 - \sum_{j=\neq i} p^*_{i,j}$, and $0 \cdot \ln 0$ is taken to be 0.

ProbOfStr: Probability of MFE structure S_0, defined by

$$\frac{\exp(-E(S_0,\mathbf{s})/RT}{Z} \tag{9}$$

where

Struct.Div: Structural diversity as defined in the source code of `RNAfold` 1.8.5, given as follows:

$$SD(\mathbf{s}) = \sum_{S,T} P(S) \cdot P(T) \cdot d_{\mathrm{BP}}(S,T) = \sum_{i=1}^{n}\sum_{j=1}^{n} p^*_{i,j} \cdot (1 - p^*_{i,j}). \tag{10}$$

where S, T range over all secondary structures of $\mathbf{s}$. The positional version of (Vienna) structural diversity is defined by

$$SD(\mathbf{s},i) = \sum_{j=1}^{n} p^*_{i,j} \cdot (1 - p^*_{i,j}). \tag{11}$$

Struct.Div.MH: Structural diversity as defined by Morgan and Higgs [10]

$$SDMH(\mathbf{s}) = n - \sum_{i=1}^{n}\sum_{j=1}^{n} (p^*_{i,j})^2. \tag{12}$$

The positional version of Morgan-Higgs structural diversity is defined by

$$SDMH(\mathbf{s},i) = 1 - \sum_{j=1}^{n} (p^*_{i,j})^2. \tag{13}$$

EntropyDistAll: This is the maximum discrepancy between the (full) structural positional entropy of wild type PLMVd and that of the current sequence $\mathbf{s}$, defined by

$$\max_{i=1}^{n} |H(\mathbf{s},i) - H(PLMVd,i)| \tag{14}$$

where $PLMVd$ denotes the RNA sequence of PLMVd AJ005312.1/282-335. The analogue for binary structural positional entropy is defined by

$$\max_{i=1}^{n} |H_b(\mathbf{s},i) - H_b(PLMVd,i)| \tag{15}$$

EBPDDistAll: This is the maximum discrepancy between expected expected base pair distance of wild type PLMVd and that of the current sequence **s**, defined by

$$\max_{i=1}^{n} |EBPD(\mathbf{s}, i)/2 - EBPD(PLMVd, i)/2| \tag{16}$$

where $PLMVd$ denotes the RNA sequence of PLMVd AJ005312.1/282-335. Division by 2 in equation (16) occurs, since values are counted twice (it was earlier mentioned that $EBPD(\mathbf{s})$ was twice the value defined in [3].

EnsDefectAll: This is the maximum discrepancy between positional ensemble defect of wild type PLMVd and that of the current sequence **s**, defined by

$$\max_{i=1}^{n} |ED(\mathbf{s}, i) - ED(PLMVd, i)| \tag{17}$$

where $PLMVd$ denotes the RNA sequence of PLMVd AJ005312.1/282-335.

StructDivAll: This is the maximum discrepancy between Vienna positional structural diversity of wild type PLMVd and that of the current sequence **s**, defined by

$$\max_{i=1}^{n} |SD(\mathbf{s}, i) - SD(PLMVd, i)| \tag{18}$$

where $PLMVd$ denotes the RNA sequence of PLMVd AJ005312.1/282-335.

StructDivMHAll: This is the maximum discrepancy between Morgan-Higgs positional structural diversity of wild type PLMVd and that of the current sequence **s**, defined by

$$\max_{i=1}^{n} |SDMH(\mathbf{s}, i)/2 - SDMH(PLMVd, i)/2| \tag{19}$$

where $PLMVd$ denotes the RNA sequence of PLMVd AJ005312.1/282-335. Division by 2 in equation (19) to avoid double counting, as in equation (16).

EntropyDistActive: This is the maximum discrepancy between the full structural positional structural entropy of wild type PLMVd and that of the current sequence **s**, defined by

$$\max_{i \in AS} |H(\mathbf{s}, i) - H(PLMVd, i)| \tag{20}$$

where $PLMVd$ denotes the RNA sequence of PLMVd AJ005312.1/282-335, and where AS designates the positions in the 'conserved site', here defined to be the following 16 positions of PLMVd: 6-8, 22-25, 27-29, 44-49. In summary, this value is given by the restriction of equation (14) to the conserved site. The analogue for binary structural positional entropy is given by

$$\max_{i \in AS} |H_b(\mathbf{s}, i) - H_b(PLMVd, i)| \tag{21}$$

EBPDDistActive: Restriction of equation (16) to the conserved site; i.e.

$$\max_{i \in AS} |EBPD(\mathbf{s}, i)/2 - EBPD(PLMVd, i)/2| \tag{22}$$

EnsDefectActive: Restriction of equation (17) to the conserved site, i.e.

$$\max_{i \in AS} |ED(\mathbf{s}, i) - ED(PLMVd, i)| \tag{23}$$

StructDivActive: Restriction of equation (18) to the conserved site; i.e.

$$\max_{i \in AS} |SD(\mathbf{s}, i) - SD(PLMVd, i)| \tag{24}$$

StructDivMHActive: Restriction of equation (19) to the conserved site; i.e.

$$\max_{i \in AS} |SDMH(\mathbf{s}, i)/2 - SDMH(PLMVd, i)/2| \tag{25}$$

3 Discussion concerning 166 nt designed ribozyme

Recently, by using an *in silico* selection pipeline employing `RNAfold` from the Vienna RNA Package, a synthetic theophylline riboswitch was rationally designed to *transcriptionally* regulate the expression of a gene [14].

As displayed in Figure 4 of the main text, in designing a hammerhead ribozyme to be a modular portion of a larger RNA molecule, for the target secondary structure S, we took the structure of the gene OFF XPT riboswitch, depicted in Figure 1A of [12], modified by replacing the terminator loop (expression platform) by the Rfam consensus structure for a type III hammerhead. As input, `RNAiFold` was given the target structure S, with the sequence constraints of Figure 4 of the main text, together with the structural constraint that all returned sequences must be compatible with a partial structure S' which sequesters the putative GUC cleavage site at positions 114-116. From the sequences returned by `RNAiFold`, we then chose a single candidate `s` for the cleavage assay, selected according to the criteria that `RNAbor`, or its faster sequel `FFTbor`, determined pronounced peaks of the density of states curve at $k = 0$, corresponding to the location of the MFE structure S, and at $k \gg 0$, corresponding to a structure T containing the base pairs in S', hence sequestering the ribozyme cleavage site NUH, located at position 114-116. Figure 3 displays the density of states as returned by `RNAbor` for the selected sequence. In this case, we did not apply the other previously discussed criteria, such as positional entropy, ensemble defect, etc.

In summary, by employing `RNAiFold` and `RNAbor`, we have designed by purely computational means a 166 nt RNA sequence `s` which contains a synthetic *functional* hammerhead ribozyme within the terminal stem-loop. Moreover, kinetics of cleavage for this artificial ribozyme are as fast as that of wild-type hammerheads. We had hoped that `s`, intended to be a rationally designed guanine riboswitch coupled with a hammerhead located within the expression platform, would demonstrate guanine-dependent cleavage. However, kinetics assays performed in the presence and absence of guanine indicate that neither the extent of cleavage (Fmax), or the rate of reaction (Kobs) changes upon addition of guanine. Figure 4A shows that while cleavage occurs in the expected location of this RNA, the kinetics of cleavage are not significantly different (Figure 4D).

The MFE and MFE(68) structures S_0 resp. S_1 of the 166 nt sequence `s` are displayed in Figure 3, where the GUC hammerhead cleavage site at position 114-116 is not sequestered [resp. is sequestered] within S_0 [resp. S_1]. The free energy of S_0 [resp. S_1], with respect to Turner 1999 energy parameters, is -95.1 [resp. -94.1] kcal/mol, and the probability of structure S_0 [resp. S_1] in the Boltzmann ensemble is 8.15% [resp. 1.61%]. The density of states graph in Figure 3 suggests that the energy landscape of the low energy ensemble could possibly be dominated by S_0, S_1, with most low energy structures resembling either S_0 or S_1. It follows that if we assume a 2-state model, then the equilibrium constant $K(S_0, S_1) = \exp(-(E(S_0) - E(S_1))/RT) \approx 5.06$; i.e. the active, cleaving form of the molecule appears five times more often than the inactive form. Moreover, the *shape* [5] of MFE structure S_0 is $\sigma_0 =$ `[[][[[][]][]]]`, while that of the MFE(68) structure S_1 is $\sigma_1 =$ `[[][]][][[][]]`. The Boltzmann probability of shape σ_0 is 98.56512%, while that of σ_1 is equal to 0.00461%, as computed by `RNAshapes` [13]; i.e. very few low energy structures have the same shape as that of the non-cleaving structure S_1. Assuming a 2-state model with states σ_0 and $\overline{\sigma_0}$, where $\overline{\sigma_0}$ denotes the complement of σ_0, we obtain an equilibrium constant of $K(\sigma_0, \overline{\sigma_0}) = 68.69$. Finally, using `RNAfold` 1.8.5 with flags `-p0 -d2 -C` and a constraint mask of `xxx` [resp. |||] at cleavage site 114-116, we determine that the ensemble free energy of structures that base-pair [resp. are unpaired] at positions 114-116 is -94.92 [resp. -94.44] kcal/mol, leading to $K(\text{unpaired}, \text{paired})$ of 2.18. These admittedly speculative computational considerations suggest that the molecule is more often in the cleavage form than not, providing a rationale for the lack of guanine-dependence seen in the cleavage data for the 166 nt designed sequence.

4 Supplemental Experimental Methods

To confirm the cleavage site of the 166 nt combined guanine riboswitch hammerhead, we mapped the sequences of the $5'$ and $3'$ cleavage products using a T1-RNase digest. RNA was transcribed *in vitro* as described in the experimental methods in the absence of the blocking peptide. The cleavage products were purified by denaturing PAGE (10% acrylamide). To generate $5'$ P32 labeled RNA, 10 pmol of RNA was dephosphorylated (alkaline phosphatase, Roche) according to the manufacturer's instructions, the phosphatase was heat inactivated, and the RNA labeled using polynucleotide kinase (NEB) and 40 μCi of γ-32P-ATP. $5'$-labeled RNA was incubated with T1-RNase (Roche) in 25 mM sodium citrate pH 5.0, 8 M urea, 1 mM

EDTA, 10% sucrose for 20 minutes at 55°C and placed on ice until gel-loading. A partial alkaline digest of the 5′ labeled RNA was performed by incubating the reaction in 50 mM $NaHCO_3$, pH 9.2 at 95°C for 5 minutes. This reaction was quenched using 2x loading buffer (16 M urea, 10 mM EDTA, 20% sucrose, 0.1%SDS, 100 mM tris pH 8.0, 100 mM borate, 0.05% bromophenol blue) , and stored on ice until gel-loading. The digestion products were separated by denaturing PAGE (10% acrylamide). The gels were dried prior to exposure to a phosphorimager screen overnight (GE Healthcare). The phosphorimager screen was scanned using a Storm 820 phosphorimager scanner to produce the images. Figure 4A and B shows that the cleavage products are consistent with the expected cleavage site of C116.

5 Dependence on parameters and algorithm

In this section, we investigate consider the dependence of our pipeline on sequence indentity threshold and on choice of RNA design algorithm.

5.1 Dependence on sequence identity threshold

In running the software `RNAiFold`, sequence constraints were imposed for those positions in the Rfam seed alignment of PLMVd type III hammerhead, for which sequence identity exceeded 96%. Subsequently, ten hammerhead candidates were selected according to various criteria concerning either (1) structural diversity or (2) matching the structural flexibility/stability of the wild type structure. What is the dependence of this protocol on the sequence identity threshold used to set constraints?

To answer this question, we ran `RNAiFold` to generate RNA sequences that (1) fold into the target structure of PLMVd, (2) have GC-content ranging from 35-55% (GC-content of wild type is 45%), and (3) have the same nucleotides in all positions whose sequence conservation in the Rfam seed alignment exceed either 90% (251,537 solutions), 96% (324,203 solutions) and 98% (349,508 solutions). We analyzed the three sets of solutions with respect to all the measures considered in the paper, but here present only a few sample figures – see Figures 6 20 and 21 as well as `http://bioinformatics.bc.edu/clotelab/SyntheticHammerheads/`.

By increasing the conservation threshold from 96% to 98%, 11 positions are constrained, rather than 15 (plus H8 constraint), and by decreasing the conservation threshold from 96% to 90%, 19 positions are constrained – see SI Table 1. Note that by design, any position, which is not constrained to be a particular nucleotide, is nevertheless constrained to be *different* than the nucleotide present in wild type PLMVd (as explained in the main text, this is to prevent selection of sequences which happen to have more sequence identity than defined).

Clearly, by adding more nucleotide constraints (e.g. decreasing the threshold from 96% to 90%), there is likely to be less sequence variation, hence the average sequence entropy is likely to be decreased. (Recall that all sequences fold into the correct target structure, so there is not a free range of nucleotide choices). However, structural entropy, ensemble defect and other measures of structural diversity tend to increase as more nucleotides are constrained to be identical to those in wild type PLMVd – i.e. structural diversity decreases for the ensemble of low energy structures of sequences, which less closely resemble the wild type sequence. At present, we have no convincing explanation for this phenomenon, which appears to be independent of the algorithm used to generate candidate hammerheads.

5.2 Dependence on inverse folding algorithm

In the main text, we mentioned a computational experiment performed with `NUPACK` [17]. Here, we compare `RNAiFold` with two recent RNA design algorithms. We ran each of `RNAiFold`, `RNAdesign` [6] and `IncaRNAtion` [11] for 4.4 hours given the same sequence constraints,[1] GC-content range from 35-55consensus structure for wild type PLMVd hammerhead. Only 2.57[resp. `RNAdesign`] actually folded into the target structure, hence it was necessary to add a very time consuming post-processing step, which involved running RNAfold on more than 12.3 million [resp. 3.3 million] sequences returned by `IncaRNAtion` [resp. `RNAdesign`]. The

[1] 15 constraints for positions having 96% sequence identity, H8 constraint, and remaining positions constrained to be different than those of wild type PLMVd hammerhead.

time required for `RNAdesign` was 4.32 hours, while that required for `IncaRNAtion` was 13.99 hours. The final statistics are summarized in Table 5.

When ensemble defect and full entropy values were sorted from smallest to largest value, we see that at 98% `RNAiFold` ¡ `RNAdesign` ¡ `IncaRNAtion`, at 96% `RNAdesign` ¡ `RNAiFold` ¡ `IncaRNAtion`, at 90% `RNAdesign` ¡ `IncaRNAtion` ¡ `RNAiFold`. In other words, when stipulating the fewest constraints (11 constraints at 98% threshold), `RNAiFold` sequences show the smallest structural diversity (smallest value of entropy and ensemble defect). These relations are illustrated in Figures 22, 23 and 24. See `http://bioinformatics.bc.edu/clotelab/SyntheticHammerheads/` for access to full data and figures for all 22 structural measures used in our study.

6 Summary of table and figures

Table 1 lists the top 20 most conserved positions of PLMVd AJ005312.1/282-335 with the corresponding frequency in the seed alignment of family RF00008 from Rfam 11.0. Table 5 shows the number of sequences returned by `RNAiFold`, `RNAdesign` [6] and `IncaRNAtion` [11], when each was run for 4.4 hours.

Figure 1 shows the very different MFE structures for PLMVd AJ005312.1/282-335, computed by Vienna RNA Package `RNAfold` 1.8.5 and 2.0.7. Figure 2 shows kinetics curves fitted from time course cleavage data for the 10 designed hammerhead sequences HH1-HH10, together with 95% confidence intervals.

Figure 3 shows the density of states, together with MFE and MFE(68) structures, computed by `RNAbor`. Figure 4 displays the T1-RNase mapping of the $5'$ (A) and $3'$ (B) cleavage products under denaturing conditions. Panel C shows kinetics of cleavage for the 166 nt RNA in the presence of guanine. Panel D shows these data plotted on the same axis as the kinetics of cleavage for the RNA in the absence of guanine (reproduced in red from Figure 8 of the main text) to show that there is no guanine dependence in extent or rate of cleavage. Table 2 shows values of Pearson correlation for measures Rate(KOBS), FMAX, ln(Rate(KOBS)), GCcont, Energy, ExpBPDist, EnsDef, computed for cleavage rates of HH1-HH10. Table 3 shows values of Pearson correlation for measures Entropy, ProbOfStr, Struct.Div, Struct.Div.MH., EntropyDistAll, EBPDDistAll, EnsDefectAll, computed for cleavage rates of HH1-HH10. Table 4 shows values of Pearson correlation for measures StructDivAll, StructDivMHAll, EntropyDistActive, EBPDDistActive, EnsDefectActive, StructDivActive, StructDivMHActive, computed for cleavage rates of HH1-HH10. Figure 5 displays the sequence logo of the 10 computationally designed hammerhead ribozymes HH1-HH10. The left panel of Figure 6 displays a hammerhead ribozyme (lower structure) hybridized with *trans*-cleavage target RNA (upper structure); the right panel shows the unrealizable structure of C116G Mutant. The left panel of Figure 7 shows the target secondary structure S for modular placement of artificial hammerhead within larger RNA molecule, while the right panel shows the final 166 nt sequence selected from over 3,000 sequence returned by `RNAiFold`, all of which respect the sequence constraints and whose MFE structure is the target structure S. Figure 8 shows the full and binary positional entropy for PLMVd AJ005312.1/282-335, while Figures 9-18 show analogous values for the 10 designed hammerheads HH1-HH10.

Figures 6, 20 and 21 respectively display the ensemble defect, full structural positional entropy, and expected base pair distance discrepancy for conserved site for output from `RNAiFold`, when different thresholds of sequence indentity (90%, 96% and 98%) are applied in selecting the positions to be constrained. Figures 22, 23 and 24 display the ensemble defect for `RNAiFold`, `RNAdesign` [6] and `IncaRNAtion` [11].

References

[1] G. E. Crooks, G. Hon, J. M. Chandonia, and S. E. Brenner. Weblogo: a sequence logo generator. *Genome Res.*, 14(6):1188–1190, June 2004.

[2] R.M. Dirks, M. Lin, E. Winfree, and N.A. Pierce. Paradigms for computational nucleic acid design. *Nucleic. Acids. Res.*, 32(4):1392–1403, 2004.

[3] J.A. Garcia-Martin, P. Clote, and I. Dotu. RNAiFold: A constraint programming algorithm for RNA inverse folding and molecular design. *Journal of Bioinformatics and Computational Biology*, 2012. in press.

[4] P. P. Gardner, J. Daub, J. Tate, B. L. Moore, I. H. Osuch, S. Griffiths-Jones, R. D. Finn, E. P. Nawrocki, D. L. Kolbe, S. R. Eddy, and A. Bateman. Rfam: Wikipedia, clans and the "decimal" release. *Nucleic. Acids. Res.*, 39(Database):D141–D145, January 2011.

[5] R. Giegerich, B. Voss, and M. Rehmsmeier. Abstract shapes of RNA. *Nucleic Acids Res.*, 32(16):4843–4851, 2004.

[6] J.C. Höner zu Siederdissen, S. Hammer, I. Abfalter, I.L. Hofacker, C. Flamm, and P.F. Stadler. Computational design of RNAs with complex energy landscapes. *Biopolymers*, 99(12):1124–1136, 2013.

[7] H.A. James and I. Gibson. The therapeutic potential of ribozymes. *Blood*, 91(2):371–381, 1998. Journal of the American Society of Hematology.

[8] D.H. Matthews, J. Sabina, M. Zuker, and D.H. Turner. Expanded sequence dependence of thermodynamic parameters improves prediction of RNA secondary structure. *J. Mol. Biol.*, 288:911–940, 1999.

[9] J.S. McCaskill. The equilibrium partition function and base pair binding probabilities for RNA secondary structure. *Biopolymers*, 29:1105–1119, 1990.

[10] S.R. Morgan and P.G. Higgs. Barrier heights between ground states in a model of RNA secondary structure. *J. Phys. A: Math. Gen.*, 31:3153–3170, 1998.

[11] V. Reinharz, Y. Ponty, and J. Waldispuhl. A weighted sampling algorithm for the design of RNA sequences with targeted secondary structure and nucleotide distribution. *Bioinformatics*, 29(13):i308–i315, July 2013.

[12] A. Serganov, Y. R. Yuan, O. Pikovskaya, A. Polonskaia, L. Malinina, A. T. Phan, C. Hobartner, R. Micura, R. R. Breaker, and D. J. Patel. Structural basis for discriminative regulation of gene expression by adenine- and guanine-sensing mRNAs. *Chem. Biol.*, 11(12):1729–1741, December 2004.

[13] P. Steffen, B. Voss, M. Rehmsmeier, J. Reeder, and R. Giegerich. RNAshapes: an integrated RNA analysis package based on abstract shapes. *Bioinformatics*, 22(4):500–503, 2006.

[14] M. Wachsmuth, S. Findeiss, N. Weissheimer, P. F. Stadler, and M. Morl. De novo design of a synthetic riboswitch that regulates transcription termination. *Nucleic. Acids. Res.*, 41(4):2541–2551, February 2013.

[15] Z. Weinberg and R. R. Breaker. R2r–software to speed the depiction of aesthetic consensus RNA secondary structures. *BMC. Bioinformatics*, 12:3, 2011.

[16] T. Xia, Jr. J. SantaLucia, M.E. Burkard, R. Kierzek, S.J. Schroeder, X. Jiao, C. Cox, and D.H. Turner. Thermodynamic parameters for an expanded nearest-neighbor model for formation of RNA duplexes with Watson-Crick base pairs. *Biochemistry*, 37:14719–35, 1999.

[17] J. N. Zadeh, B. R. Wolfe, and N. A. Pierce. Nucleic acid sequence design via efficient ensemble defect optimization. *J. Comput. Chem.*, 32(3):439–452, February 2011.

Rank	Position (1-54)	Nucleotide	Frequency	Percentage
1	7	U	1	100%
2	23	U	1	100%
3	27	G	1	100%
4	22	C	1	100%
5	48	A	1	100%
6	47	A	1	100%
7	28	A	1	100%
8	25	A	1	100%
9	24	G	0.988095	99%
10	46	A	0.988095	99%
11	6	G	0.987342	99%
12	45	G	0.97619	98%
13	49	C	0.97561	98%
14	29	G	0.964286	96%
15	44	C	0.964286	96%
16	38	A	0.957746	96%
17	8	C	0.949367	95%
18	35	G	0.942857	94%
19	42	G	0.928571	93%
20	31	C	0.892857	89%

Table 1: Top 20 most conserved positions of PLMVd AJ005312.1/282-335 with the corresponding frequency in the seed alignment of family RF00008 from Rfam 11.0. Note the conservation rate of 95% for cleavage site C8.

Table 2: Pearson correlation for the following measures: observable cleavage rate Rate(KOBS), maximum proportion cleaved (FMAX), ln(Rate(KOBS)), GC-content (GCcont), free energy (Energy), expected base pair distance (Exp. BP Distance), ensemble defect (EnsDef), computed for cleavage rates of HH1-HH10.

	Rate(KOBS)	FMAX	ln(Rate(KOBS))	GC content	Free Energy	Exp. BP Distance	Ensemble Defect
Rate(KOBS)	1	0.3180495316	0.8010267171	-0.0879851913	-0.0217082614	-0.351659924	-0.3700535691
FMAX	0.3180495316	1	0.7395577848	-0.2222788422	0.0875729623	-0.2670275304	-0.2649674962
ln(Rate(KOBS))	0.8010267171	0.7395577848	1	-0.0140654147	-0.1056892951	-0.1410789885	-0.1455861136
GC content	-0.0879851913	-0.2222788422	-0.0140654147	1	-0.9559888012	0.8084253547	0.7574113301
Free Energy	-0.0217082614	0.0875729623	-0.1056892951	-0.9559888012	1	-0.6578683824	-0.5837607785
Exp. BP Dist	-0.351659924	-0.2670275304	-0.1410789885	0.8084253547	-0.6578683824	1	0.9931047909
Ensemble Defect	-0.3700535691	-0.2649674962	-0.1455861136	0.7574113301	-0.5837607785	0.9931047909	1
Full Entropy	-0.4611188265	-0.4111937577	-0.2991394055	0.6644957954	-0.4735471419	0.9616094831	0.973189489
Prob. of Structure	-0.1663986282	-0.2677070799	-0.3670590956	0.10111802	-0.2105759941	-0.0170753704	-0.0984343968
Structural Diversity	-0.3813280943	-0.3176320303	-0.193122571	0.7921564709	-0.6369883805	0.9976707899	0.9905774746
Structural Diversity (MH)	-0.3999366943	-0.300850507	-0.1843873698	0.7151441144	-0.5242172098	0.9799388933	0.9954653096
Entropy Divergence	0.0212393635	0.2228759583	0.1328306809	0.5142254913	-0.5573781302	0.3347862524	0.2755163468
EBPD Divergence	0.0991303589	0.4390072815	0.2799247817	0.0733597819	-0.1521312817	-0.0196083109	-0.0487197823
Ens. Def. Divergence	0.3426578354	0.5916515677	0.4303742692	-0.2023228875	0.0411714938	-0.4686331775	-0.4977033786
Struct. Div. Divergence	0.3736719402	0.6418100727	0.518818656	-0.1217887987	-0.0428895468	-0.3661375818	-0.3973682459
Struct. Div. (MH) Distance	0.3742035731	0.3309967218	0.1895766441	-0.4519280593	0.2231905034	-0.7887509201	-0.842392592
Entropy Divergence(CS)	-0.2028988883	0.0984735236	-0.1175962352	0.4517940169	-0.5227903496	0.2785447359	0.2032084408
EBPD Divergence(CS)	-0.4380851142	0.1218512855	-0.3214985566	-0.1067054412	0.0560142537	-0.013145995	-0.0518034311
Ens. Def. Divergence(CS)	-0.1183239032	0.2690619624	-0.0466756203	0.04234593	-0.1834279809	-0.1221119147	-0.1922427955
Struct. Div. Divergence(CS)	-0.0257136684	0.3972177731	0.1056462355	0.0512665143	-0.1946388389	-0.1219272042	-0.1829906768
Struct. Div. (MH) Divergence(CS)	0.3678806112	0.3285082582	0.1828505319	-0.4548024962	0.226642378	-0.7898712856	-0.8435525769

Table 3: Pearson correlation for the following measures: Full structural positional entropy (Entropy), Boltzmann probability of structure (ProbOfStr), Vienna structural diversity (Struct.Div.), Morgan-Higgs structural diversity (Struct.Div.MH.), discrepancy between positional entropy of wild type and of synthetic hammerhead (EntropyDistAll), discrepancy between expected base pair distance of wild type and of synthetic hammerhead (EBPDDistAll), discrepancy between ensemble defect of wild type and of synthetic hammerhead (EnsDefectAll), computed for cleavage rates of HH1-HH10.

	Full Entropy	Prob. of Structure	Structural Diversity	Structural Diversity (MH)	Entropy Divergence	EBPD Divergence	Ens. Def. Divergence
Rate(KOBS)	-0.4611188265	-0.1663986282	-0.3813280943	-0.3999366943	0.0212393635	0.0991303589	0.3426578354
FMAX	-0.4111937577	-0.2677070799	-0.3176320303	-0.300850507	0.2228759583	0.4390072815	0.5916515677
ln(Rate(KOBS))	-0.2991394055	-0.3670590956	-0.193122571	-0.1843873698	0.1328306809	0.2799247817	0.4303742692
GC content	0.6644957954	0.10111802	0.7921564709	0.7151441144	0.5142254913	0.0733597819	-0.2023228875
Free Energy	-0.4735471419	-0.2105759941	-0.6369883805	-0.5242172098	-0.5573781302	-0.1521312817	0.0411714938
Exp. BP Distance	0.9616094831	-0.0170753704	0.9976707899	0.9799388933	0.3347862524	-0.0196083109	-0.4686331775
Ensemble Defect	0.973189489	-0.0984343968	0.9905774746	0.9954653096	0.2755163468	-0.0487197823	-0.4977033786
Full Entropy	1	-0.0464704594	0.9741987559	0.982046423	0.1235798533	-0.1661460797	-0.6325565623
Prob. of Structure	-0.0464704594	1	0.014125765	-0.142246453	0.4766956636	0.3632012249	0.2188680546
Structural Diversity	0.9741987559	0.014125765	1	0.9802394435	0.3052810263	-0.0473673309	-0.5054505713
Structural Diversity (MH)	0.982046423	-0.142246453	0.9802394435	1	0.2096413628	-0.0802993511	-0.5322042806
Entropy Divergence	0.1235798533	0.4766956636	0.3052810263	0.2096413628	1	0.7467667202	0.5619693341
EBPD Divergence	-0.1661460797	0.3632012249	-0.0473673309	-0.0802993511	0.7467667202	1	0.8497064491
Ens. Def. Divergence	-0.6325565623	0.2188680546	-0.5054505713	-0.5322042806	0.5619693341	0.8497064491	1
Struct. Div. Divergence	-0.5452931363	0.218521363	-0.4066924876	-0.4383602277	0.601857192	0.8852325901	0.9848011755
Struct. Div. (MH) Distance	-0.8756138699	0.4825886185	-0.7943770035	-0.8846688676	0.2112810754	0.3225100507	0.6735719477
Entropy Divergence(CS)	0.0992499995	0.6376456907	0.2645214219	0.139085458	0.9312030661	0.6504101932	0.4824535979
EBPD Divergence(CS)	-0.0560891157	0.6819688623	-0.0036814596	-0.077930885	0.6205236921	0.6547856326	0.4805790098
Ens. Def. Divergence(CS)	-0.2681432854	0.689164349	-0.1319123991	-0.2461161305	0.782370952	0.7685421115	0.7219357942
Struct. Div. Divergence(CS)	-0.2912050548	0.6029252375	-0.1425295001	-0.2389607782	0.8235464729	0.8622609177	0.8173588398
Struct. Div. (MH) Divergence(CS)	-0.8757857638	0.4841139947	-0.7951938049	-0.8854498404	0.2112647608	0.3232436256	0.673827166

Table 4: Pearson correlation for the following measures: discrepancy between Vienna structural diversity of wild type and of synthetic hammerhead (StructDivAll), discrepancy between Morgan-Higgs structural diversity of wild type and of synthetic hammerhead (StructDivMHAll), discrepancy between positional entropy of wild type and of synthetic hammerhead both restricted to the conserved site (EntropyDist CS), discrepancy between expected base pair distance of wild type and of synthetic hammerhead both restricted to the conserved site (EBPDDist CS), discrepancy between ensemble defect of wild type and of synthetic hammerhead both restricted to the conserved site (EnsDefect CS), discrepancy between Vienna structural diversity of wild type and of synthetic hammerhead both restricted to the conserved site (StructDiv CS), discrepancy between Morgan-Higgs structural diversity of wild type and of synthetic hammerhead both restricted to the conserved site (StructDivMH CS), computed for cleavage rates of HH1-HH10.

	Struct. Div. Divergence	Struct. Div. (MH) Distance	Entropy Divergence(CS)	EBPD Divergence(CS)	Ens. Def. Divergence(CS)	Struct. Div. Divergence(CS)	Struct. Div. (MH) Divergence(CS)
Rate(KOBS)	0.3736719402	0.3742035731	-0.2028988883	-0.4380851142	-0.1183239032	-0.0257136684	0.3678806112
FMAX	0.6418100727	0.3309967218	0.0984735236	0.1218512855	0.2690619624	0.3972177731	0.3285082582
ln(Rate(KOBS))	0.518818656	0.1895766441	-0.1175962352	-0.3214985566	-0.0466756203	0.1056462355	0.1828505319
GC content	-0.1217887987	-0.4519280593	0.4517940169	-0.1067054412	0.04234593	0.0512665143	-0.4548024962
Free Energy	-0.0428895468	0.2231905034	-0.5227903496	0.0560142537	-0.1834279809	-0.1946388389	0.226642378
Exp. BP Distance	-0.3661375818	-0.7887509201	0.2785447359	-0.013145995	-0.1221119147	-0.1219272042	-0.7898712856
Ensemble Defect	-0.3973682459	-0.842392592	0.2032084408	-0.0518034311	-0.1922427955	-0.1829906768	-0.8435525769
Full Entropy	-0.5452931363	-0.8756138699	0.0992499995	-0.0560891157	-0.2681432854	-0.2912050548	-0.8757857638
Prob. of Structure	0.218521363	0.4825886185	0.6376456907	0.6819688623	0.689164349	0.6029252375	0.4841139947
Structural Diversity	-0.4066924876	-0.7943770035	0.2645214219	-0.0036814596	-0.1319123991	-0.1425295001	-0.7951938049
Structural Diversity (MH)	-0.4383602277	-0.8846688676	0.139085458	-0.077930885	-0.2461161305	-0.2389607782	-0.8854498404
Entropy Divergence	0.601857192	0.2112810754	0.9312030661	0.6205236921	0.782370952	0.8235464729	0.2112647608
EBPD Divergence	0.8852325901	0.3225100507	0.6504101932	0.6547856326	0.7685421115	0.8622609177	0.3232436256
Ens. Def. Divergence	0.9848011755	0.6735719477	0.4824535979	0.4805790098	0.7219357942	0.8173588398	0.673827166
Struct. Div. Divergence	1	0.6112095695	0.4917123078	0.456847097	0.7039316548	0.8194681669	0.6101196512
Struct. Div. (MH) Distance	0.6112095695	1	0.2846925162	0.3599941844	0.5860495242	0.5788515268	0.9999298109
Entropy Divergence(CS)	0.4917123078	0.2846925162	1	0.7725730573	0.8884751252	0.861084344	0.2876460557
EBPD Divergence(CS)	0.456847097	0.3599941844	0.7725730573	1	0.8899628696	0.8370687809	0.366294153
Ens. Def. Divergence(CS)	0.7039316548	0.5860495242	0.8884751252	0.8899628696	1	0.9765305455	0.5902199926
Struct. Div. Divergence(CS)	0.8194681669	0.5788515268	0.861084344	0.8370687809	0.9765305455	1	0.5812616731
Struct. Div. (MH) Divergence(CS)	0.6101196512	0.9999298109	0.2876460557	0.366294153	0.5902199926	0.5812616731	1

	`RNAiFold`	`RNAdesign`	`IncaRNAtion`
Tot seq	180,243	3,382,729	12,332,554
% fold to target	100.00%	5.84%	2.57%
Final num seq	180243	197414	317218

Table 5: Summary statistics on number of sequences generated by `RNAiFold`, `RNAdesign`, and `IncaRNAtion` within 4.4 hours, together with the percentage and number of sequences that fold into the input target structure.

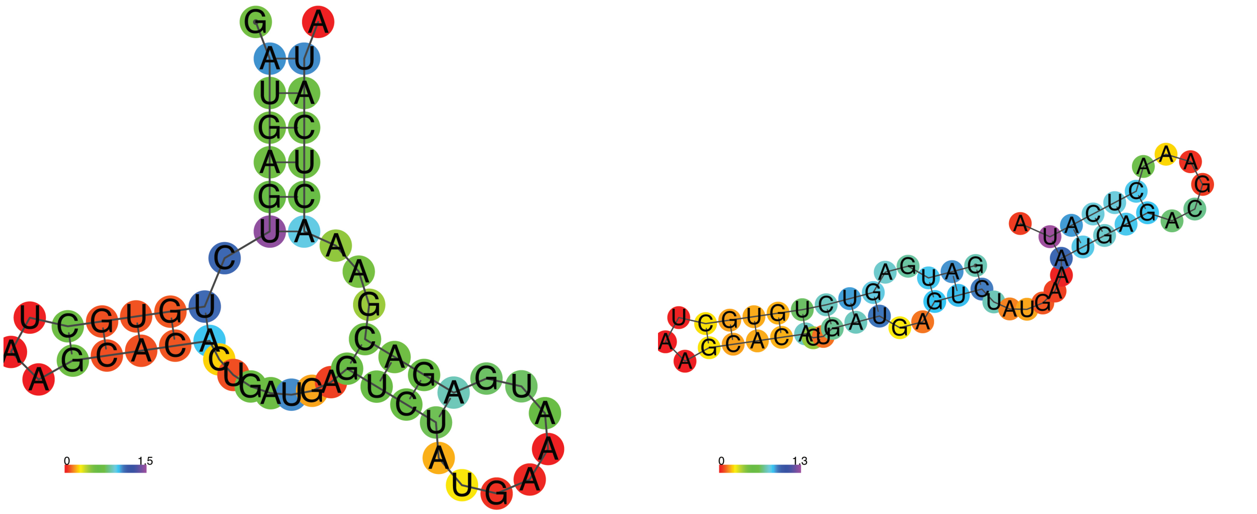

Figure 1: Predicted secondary structure of the 54 nt Peach Latent Mosaic Viroid (PLMVd) AJ005312.1/282-335, colored by (full) structural positional entropy. (*Left*) Vienna RNA Package 1.8.5, employing the Turner 1999 free energy parameters. (*Right*) Vienna RNA Package 2.0.7, employing the Turner 2004 free energy parameters. Since the Vienna 1.8.5 MFE structure is identical to the Rfam consensus structure of PLMVd AJ005312.1/282-335, we used `RNAiFold 1.8.5` rather than `RNAiFold 2.0.7` for this work.

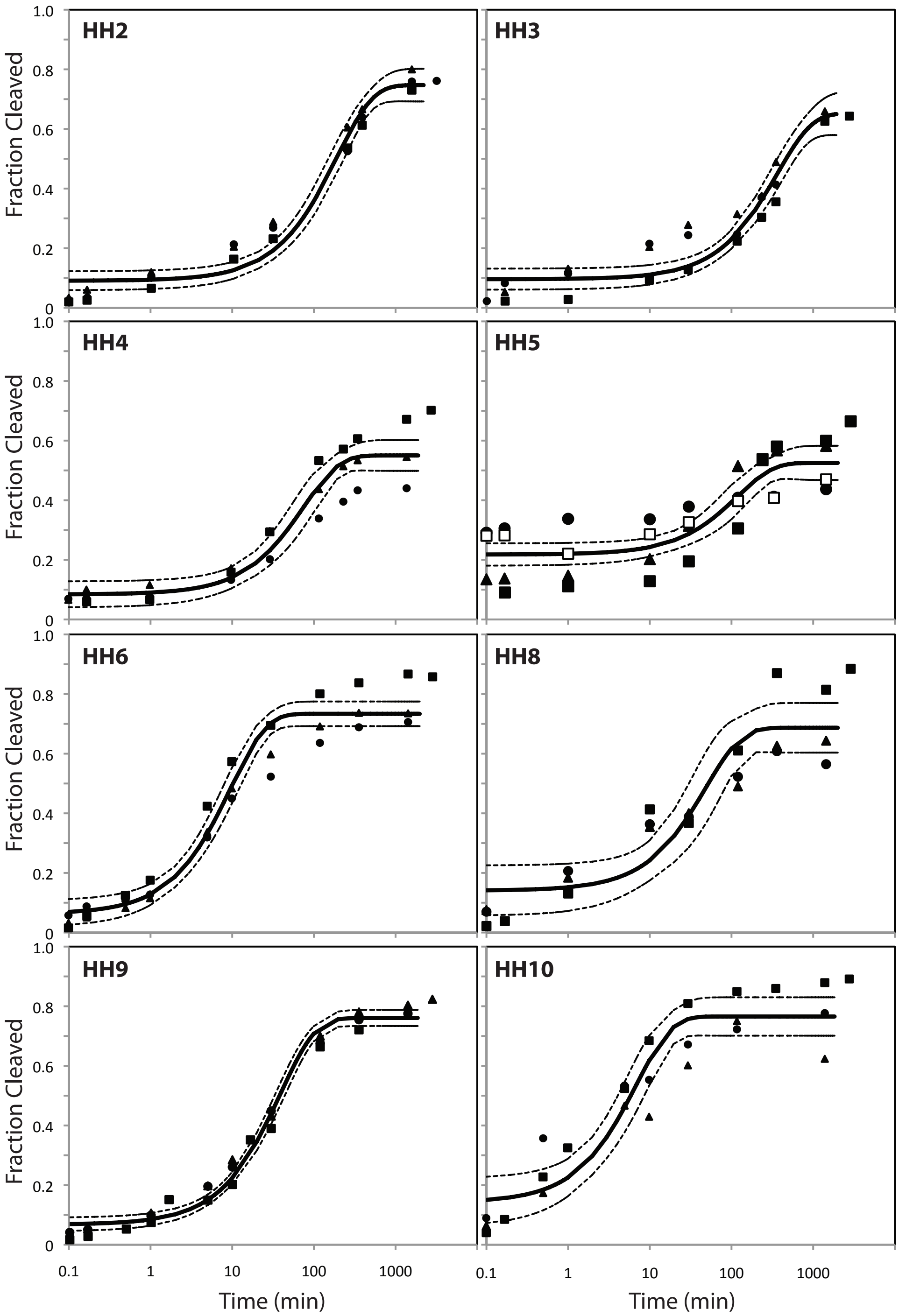

Figure 2: Best-fit kinetics curves for designed hammerhead sequences (see Methods in main text). From three to five independent replicates of the time series were conducted for each designed hammerhead sequence. Each series is represented by a marker of different shape (e.g. closed square, closed circle). The solid line represents the best-fit curve, and the dotted lines represent the 95% confidence interval (see Methods in the main text for details on the calculation). The curves for HH1 and HH7 are displayed in the main text and are not repeated here.

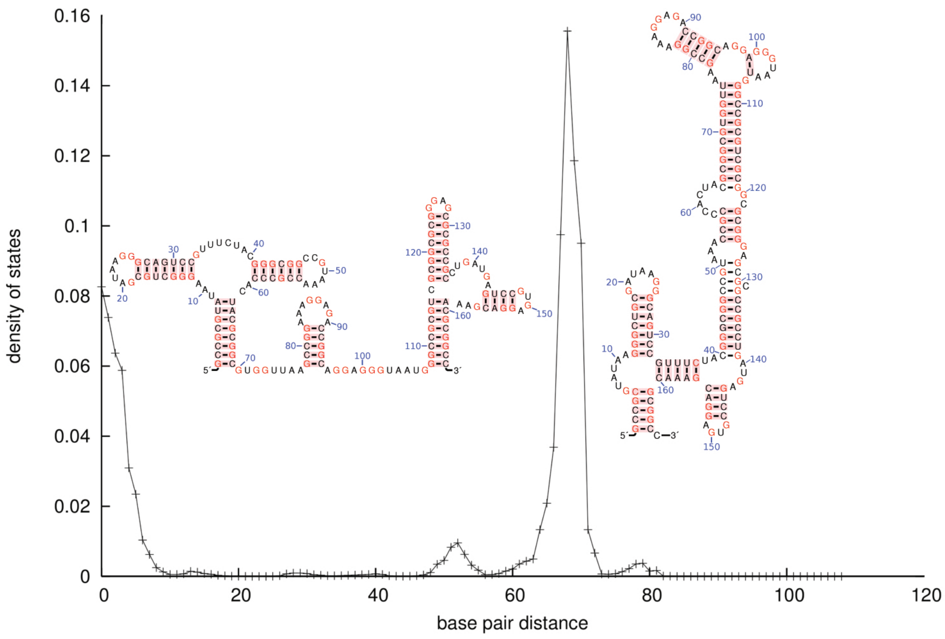

Figure 3: Density of states graph, computed by `RNAbor`, together with the MFE structure S and the MFE_{68} structure, found respectively at the left and right peaks. Metastable structure MFE_{68} contains all base pairs from compatibility constraint S', thus in theory sequestering the GUC hammerhead cleavage site at position 114-116. Density of states and secondary structures produced by `RNAbor`; superimposed secondary structure images produced by R2R [15].

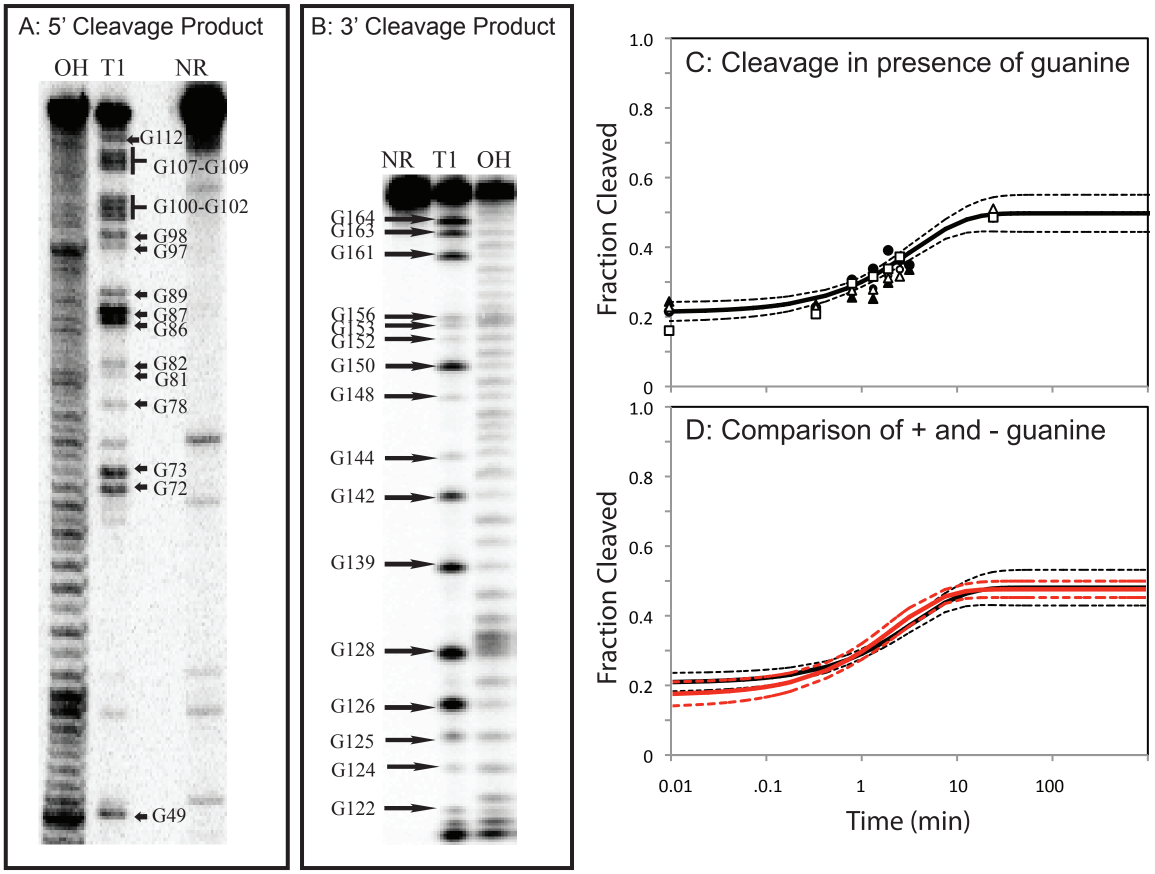

Figure 4: A: T1-RNase mapping of the 5′ cleavage product under denaturing conditions. Lane 1 is the partial alkaline digest (OH), followed by the RNAse T1 digest (T1), and the undigested RNA (NR). B: T1-RNAse mapping of the 3′ cleavage product. Lane 1 is the undigested RNA (NR) followed by the RNAse T1 digest (T1), and the partial alkaline digest (OH). The T1 digest of both the 5′ cleavage product and the 3′ cleavage product are consistent with the predicted cleavage position at C116. C: Cleavage kinetics for 166 nt RNA in the presence of 1 mM guanine. Five independent time courses were conducted and the best-fit curve and 95% confidence interval (dashed lines) determined as described in the Methods in the main text. D: The best-fit curves and 95% confidence interval of the cleavage in the presence (black) and absence (red) of guanine plotted onto the same set of axis. Cleavage in the absence of guanine is reproduced from Figure 7 in the main test.

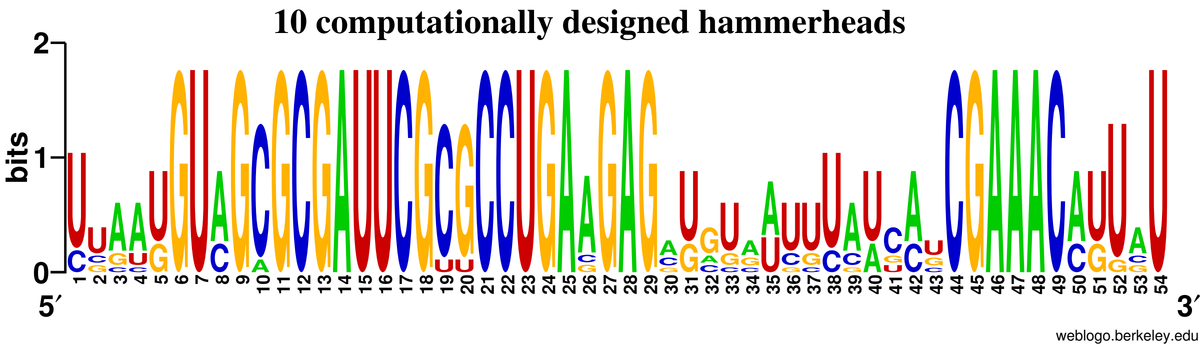

Figure 5: Logo of the 10 computationally designed hammerhead ribozymes. These sequences were selected from over one million 54 nt sequences returned by `RNAiFold`, each of whose minimum free energy structure is the target Rfam consensus structure of Peach Latent Mosaic Viroid (PLMVd) AJ005312.1/282-335. Moreover, all sequences returned by `RNAiFold` satisfied the sequence constraints `HBVHBGUHVH VHDVBBHDBD BCUGAVGAGV DVBVHBBBVH BHBCGAAACV DBVB`. Sequence logo produced by Weblogo [1].

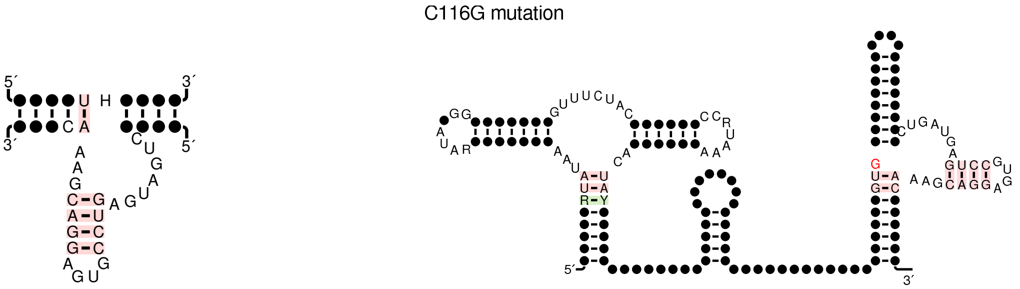

Figure 6: (*Left*) Hammerhead ribozyme (lower molecule) hybridized with *trans*-cleavage target RNA (upper molecule). Undetermined nucleotides are designated by • in the figure, and denoted by 'N' in text, while cleavage site is indicated by IUPAC code NUH. `RNAiFold` shows that no two sequences $\mathbf{s}_1, \mathbf{s}_2$ exist, where $\mathbf{s}_1$ contains 'GUG' at positions 4-6, both $\mathbf{s}_1, \mathbf{s}_2$ contain the other indicated nucleotides, for which the indicated structure is the MFE hybridization of $\mathbf{s}_1, \mathbf{s}_2$. Image adapted from Figure 3A from [7]. (*Right*) Unrealizable structure of C116G Mutant. Red G at position 116 in the sequence is the mutated nucleotide. As earlier mentioned, `RNAiFold` determined that no sequence exists, whose MFE structure is that of PLMVd AJ005312.1/282- 335, which has the nucleotides of PLMVd at the 15 positions 6-7, 22-25,27-29, 44-49, together with a guanine at cleavage position 8. Taken together, these results strongly suggest that there are structural reasons that explain why experimentalist have only observed cleavage with the hammerhead motif NUH.

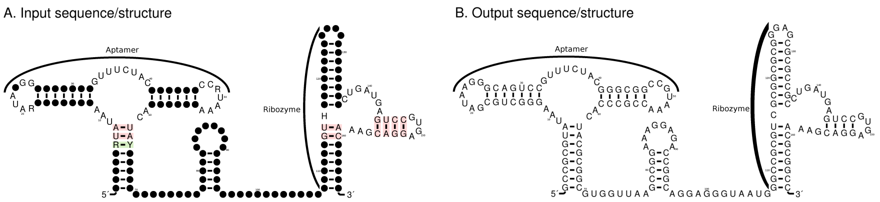

Figure 7: *(Left)* Target secondary structure S for modular placement of artificial hammerhead within larger RNA molecule. The structure and highly conserved nucleotides (sequence constraints) of the XPT-riboswitch appear on the left, while the structure and highly conserved nucleotides of the type III hammerhead ribozyme appear on the right. *(Right)* Sequence $\mathbf{s}$ filtered using `RNAbor` from over 3,000 sequence returned by `RNAiFold`, all of which respect the sequence constraints and whose MFE structure is the target structure S.

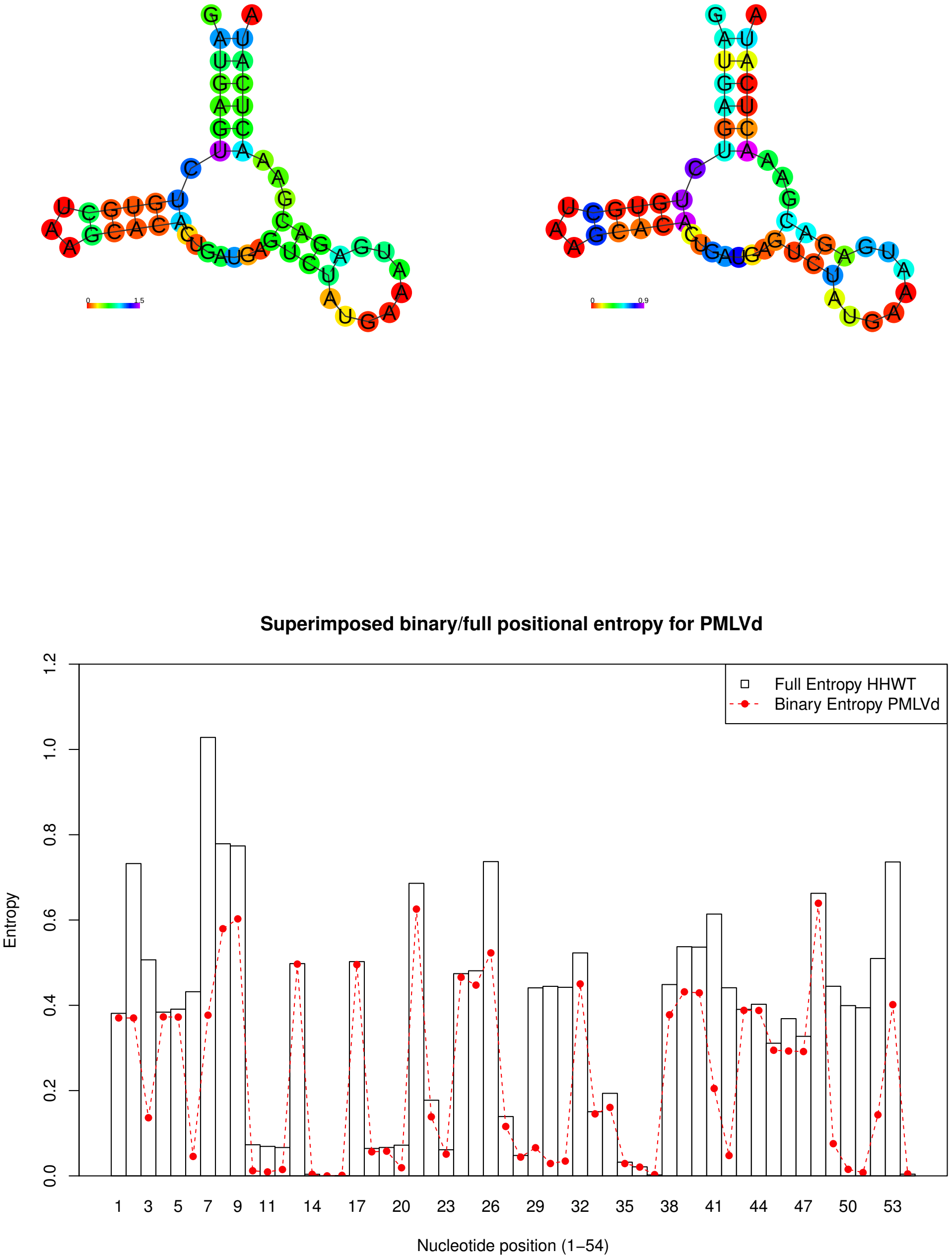

Figure 8: *(Left)* Full structural positional entropy of Peach Latent Mosaic Viroid (PLMVd) AJ005312.1/282-335. *(Right)* Binary structural positional entropy of Peach Latent Mosaic Viroid (PLMVd) AJ005312.1/282-335. *(Down)* Overlay of full and binary structural positional entropy of Peach Latent Mosaic Viroid (PLMVd) AJ005312.1/282-335.

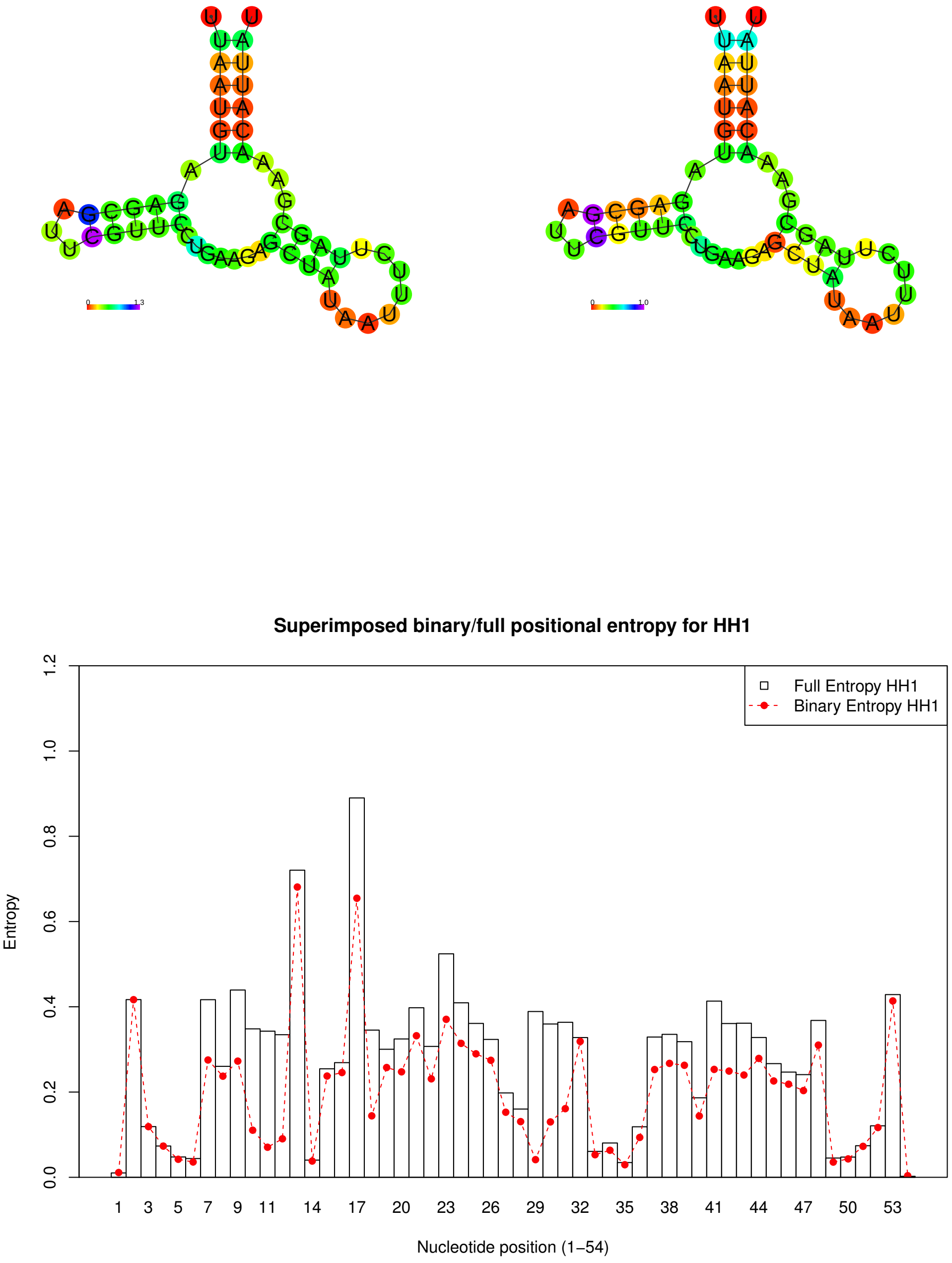

Figure 9: *(Left)* Full structural positional entropy of HH1. *(Right)* Binary structural positional entropy of HH1. *(Down)* Overlay of full and binary structural positional entropy of HH1.

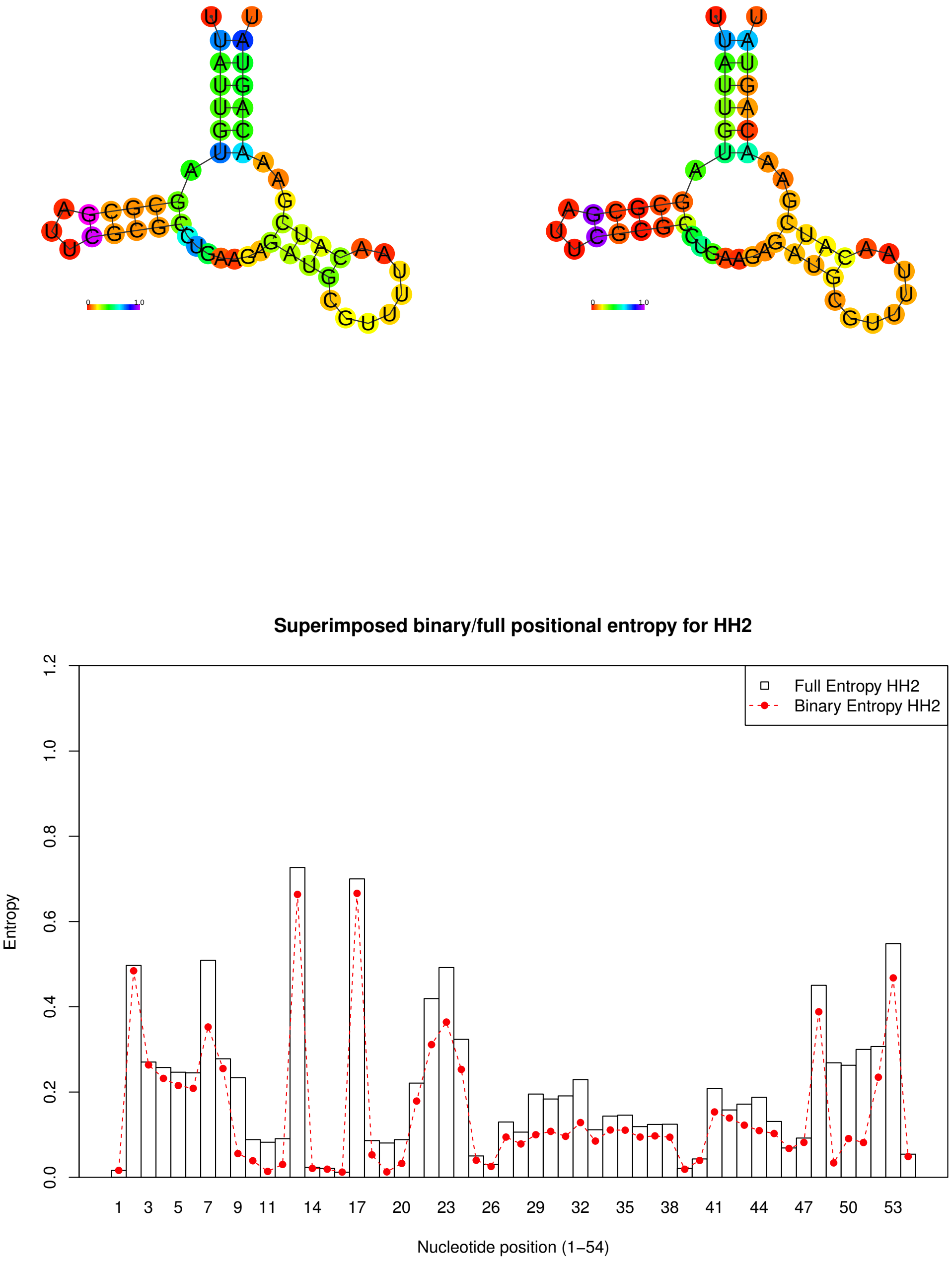

Figure 10: *(Left)* Full structural positional entropy of HH2. *(Right)* Binary structural positional entropy of HH2. *(Down)* Overlay of full and binary structural positional entropy of HH2.

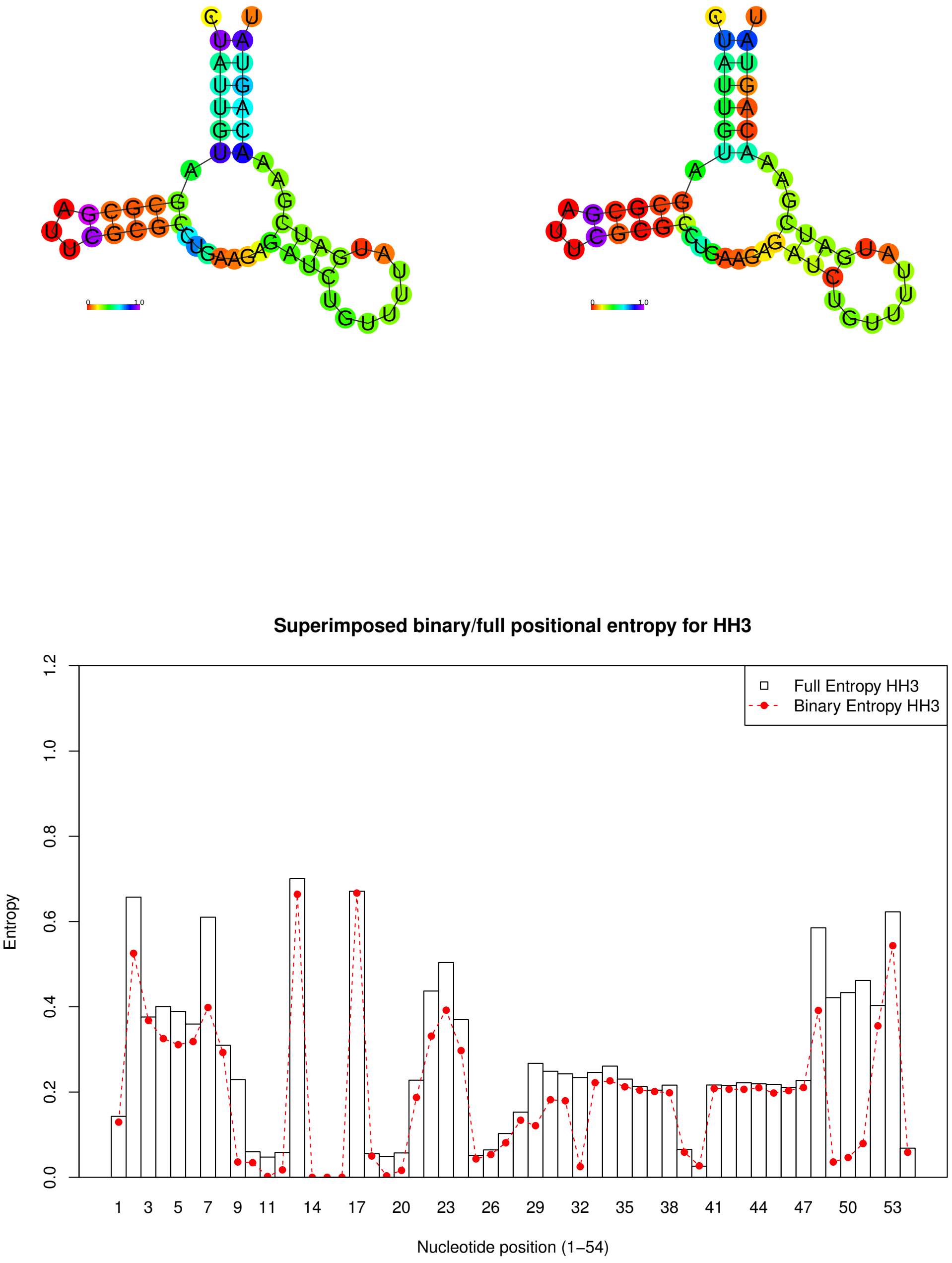

Figure 11: *(Left)* Full structural positional entropy of HH3. *(Right)* Binary structural positional entropy of HH3. *(Down)* Overlay of full and binary structural positional entropy of HH3.

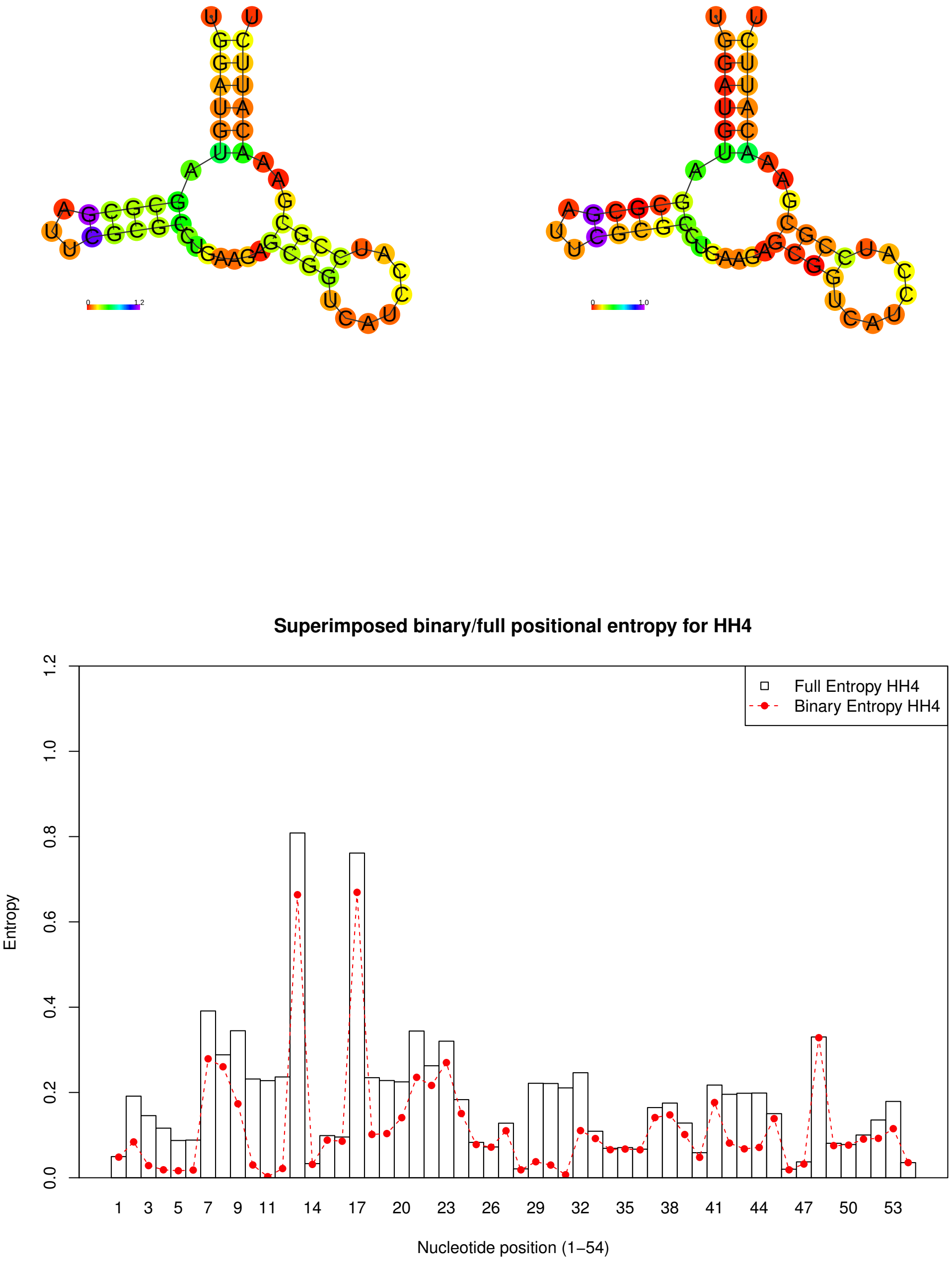

Figure 12: *(Left)* Full structural positional entropy of HH4. *(Right)* Binary structural positional entropy of HH4. *(Down)* Overlay of full and binary structural positional entropy of HH4.

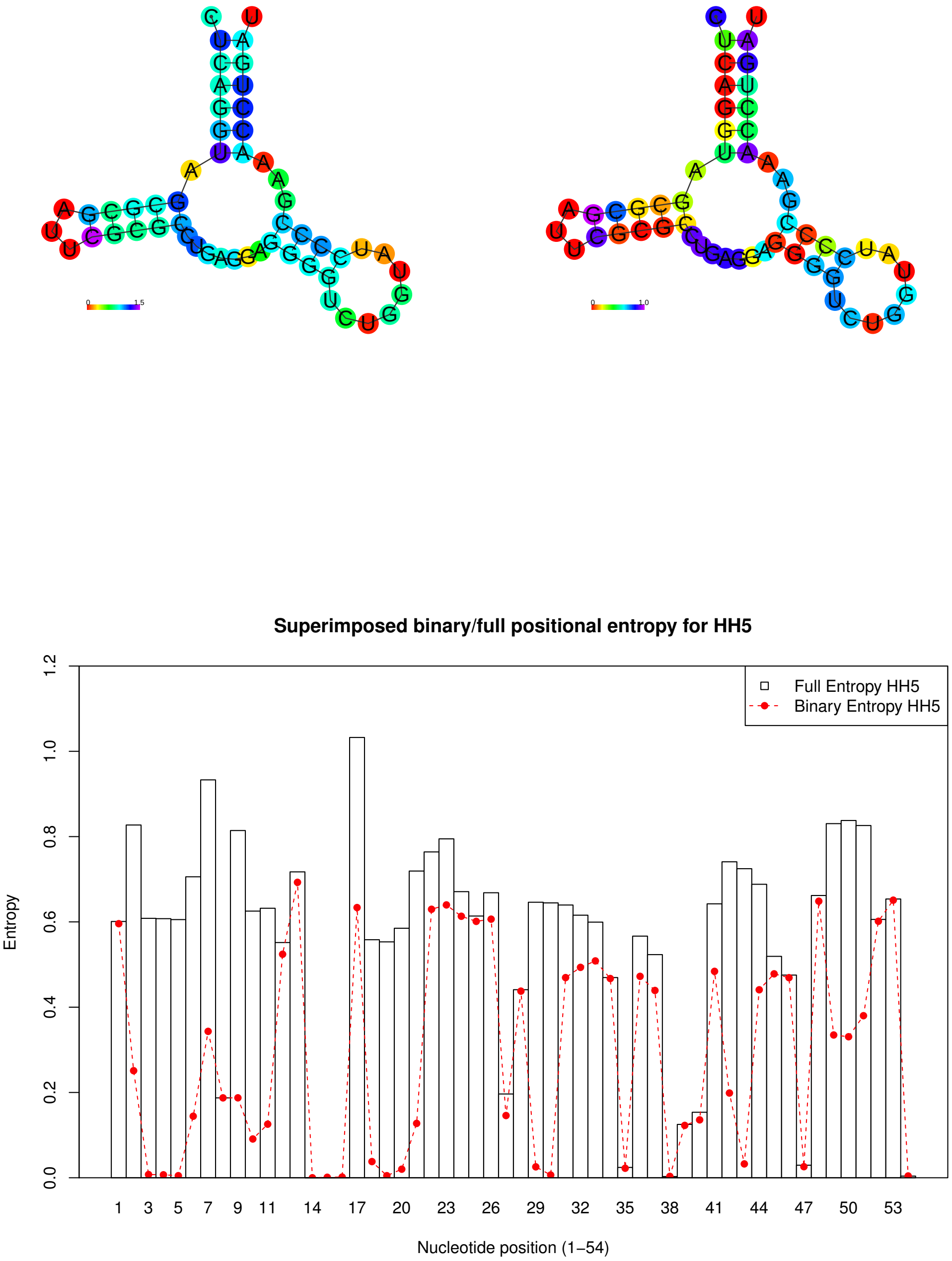

Figure 13: *(Left)* Full structural positional entropy of HH5. *(Right)* Binary structural positional entropy of HH5. *(Down)* Overlay of full and binary structural positional entropy of HH5.

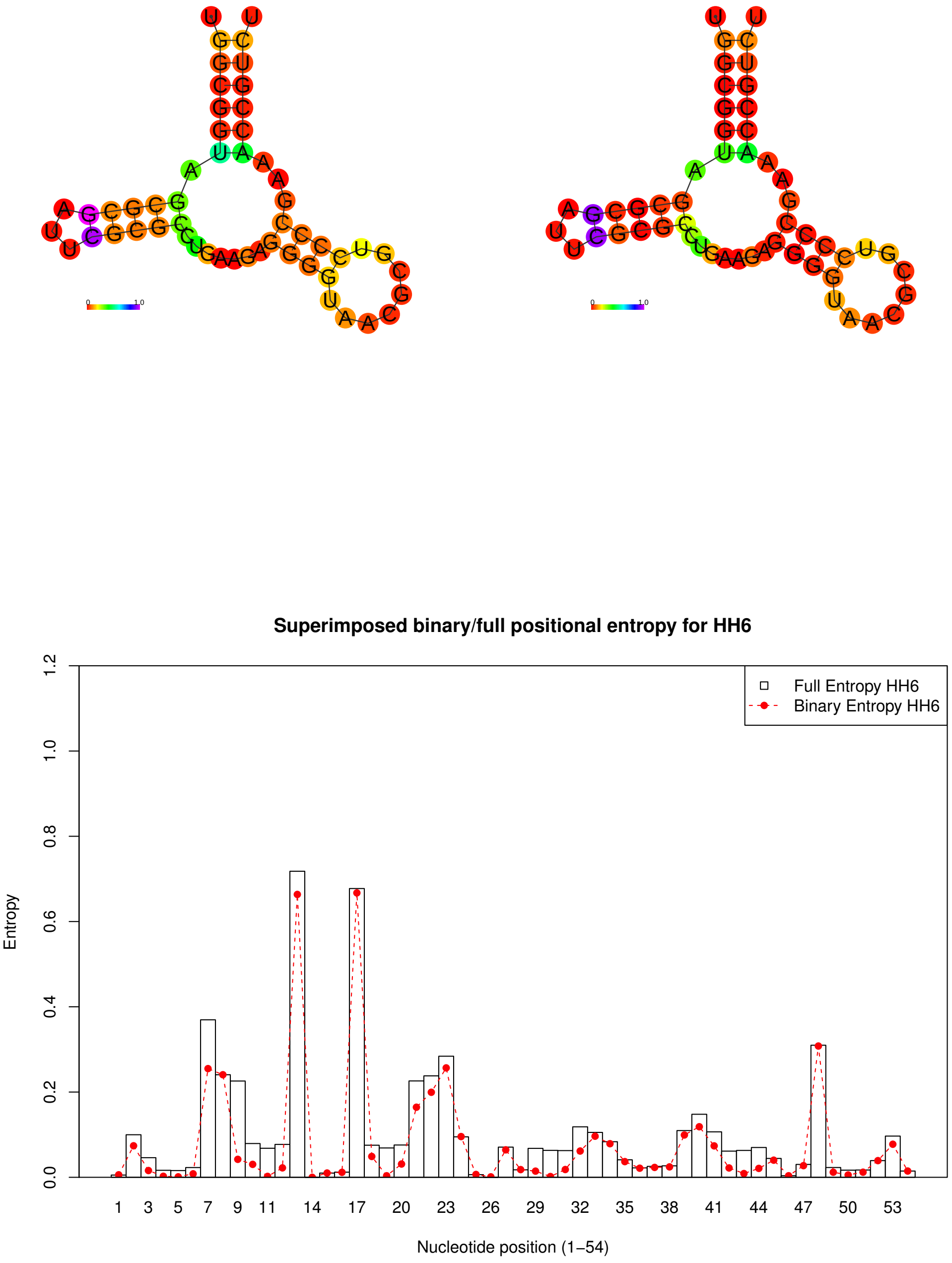

Figure 14: *(Left)* Full structural positional entropy of HH6. *(Right)* Binary structural positional entropy of HH6. *(Down)* Overlay of full and binary structural positional entropy of HH6.

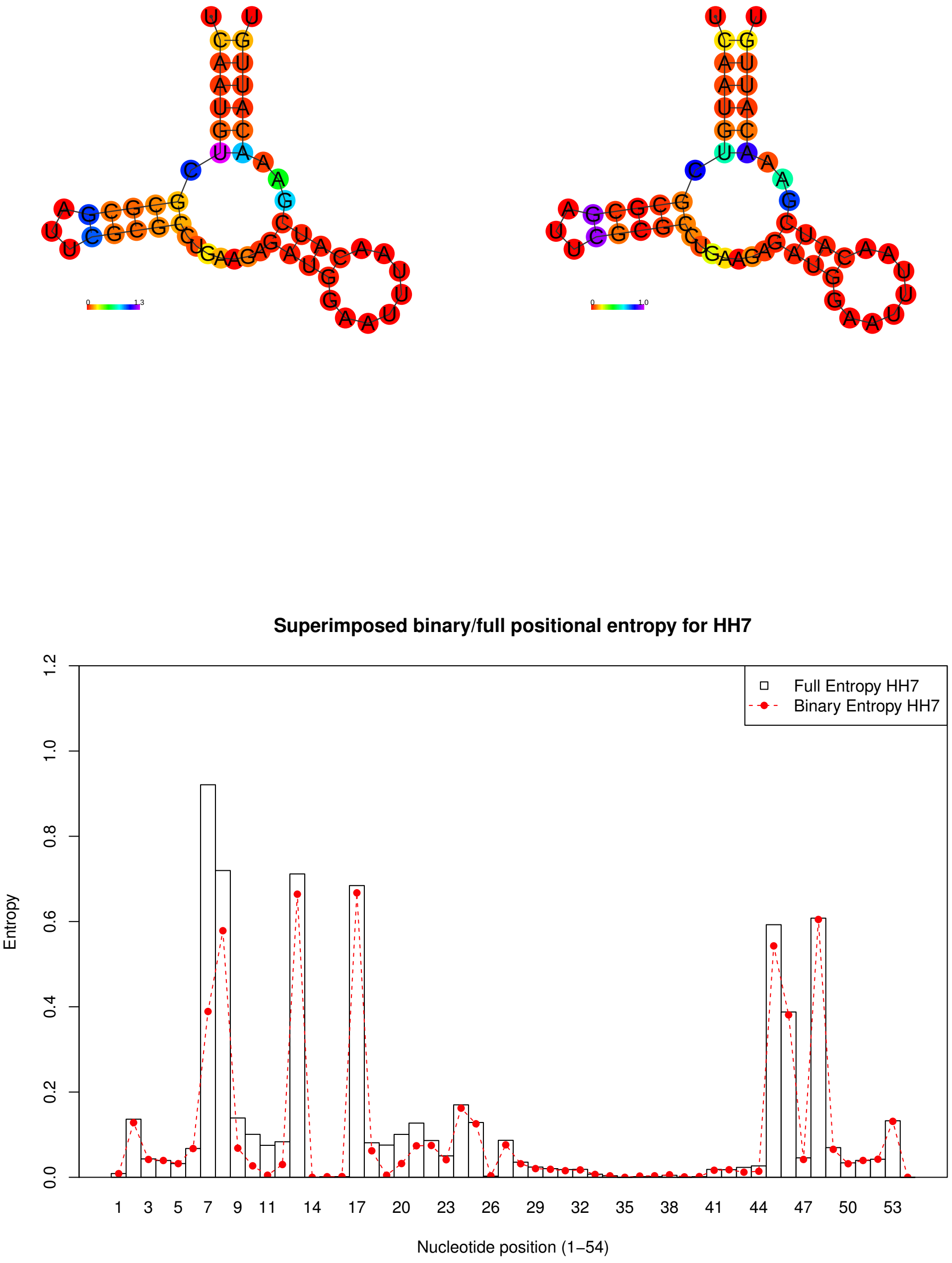

Figure 15: *(Left)* Full structural positional entropy of HH7. *(Right)* Binary structural positional entropy of HH7. *(Down)* Overlay of full and binary structural positional entropy of HH7.

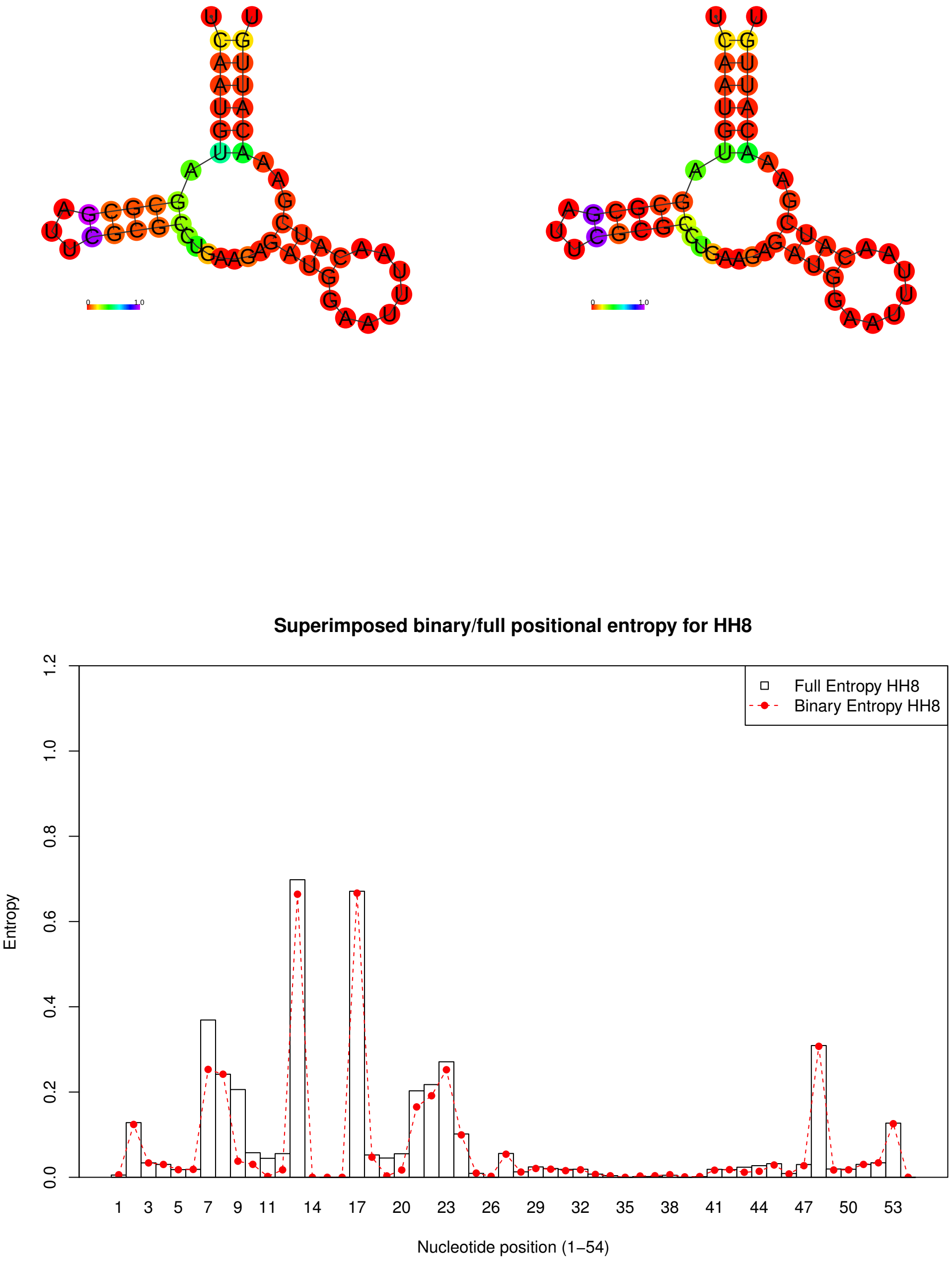

Figure 16: *(Left)* Full structural positional entropy of HH8. *(Right)* Binary structural positional entropy of HH8. *(Down)* Overlay of full and binary structural positional entropy of HH8.

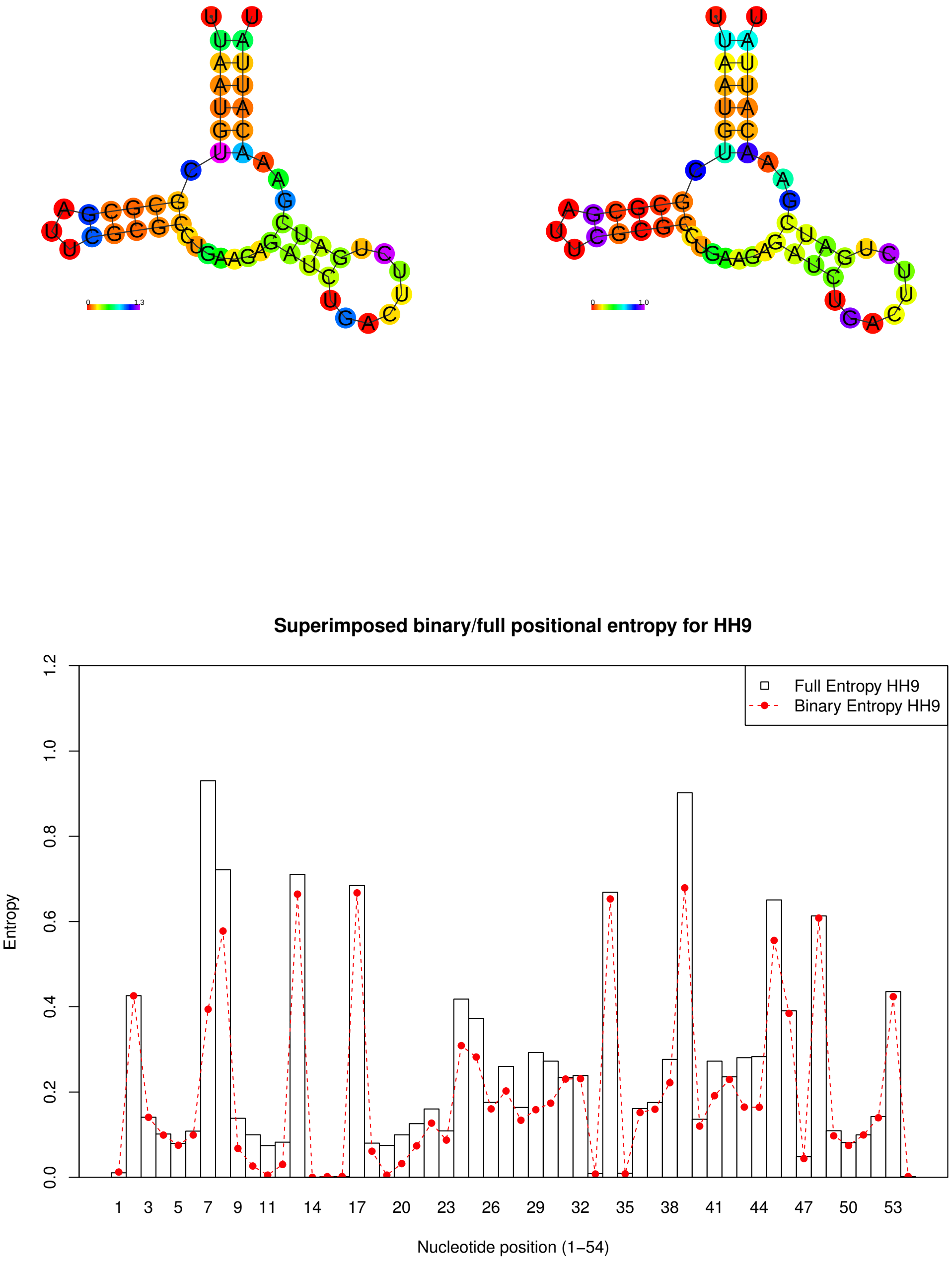

Figure 17: *(Left)* Full structural positional entropy of HH9. *(Right)* Binary structural positional entropy of HH9. *(Down)* Overlay of full and binary structural positional entropy of HH9.

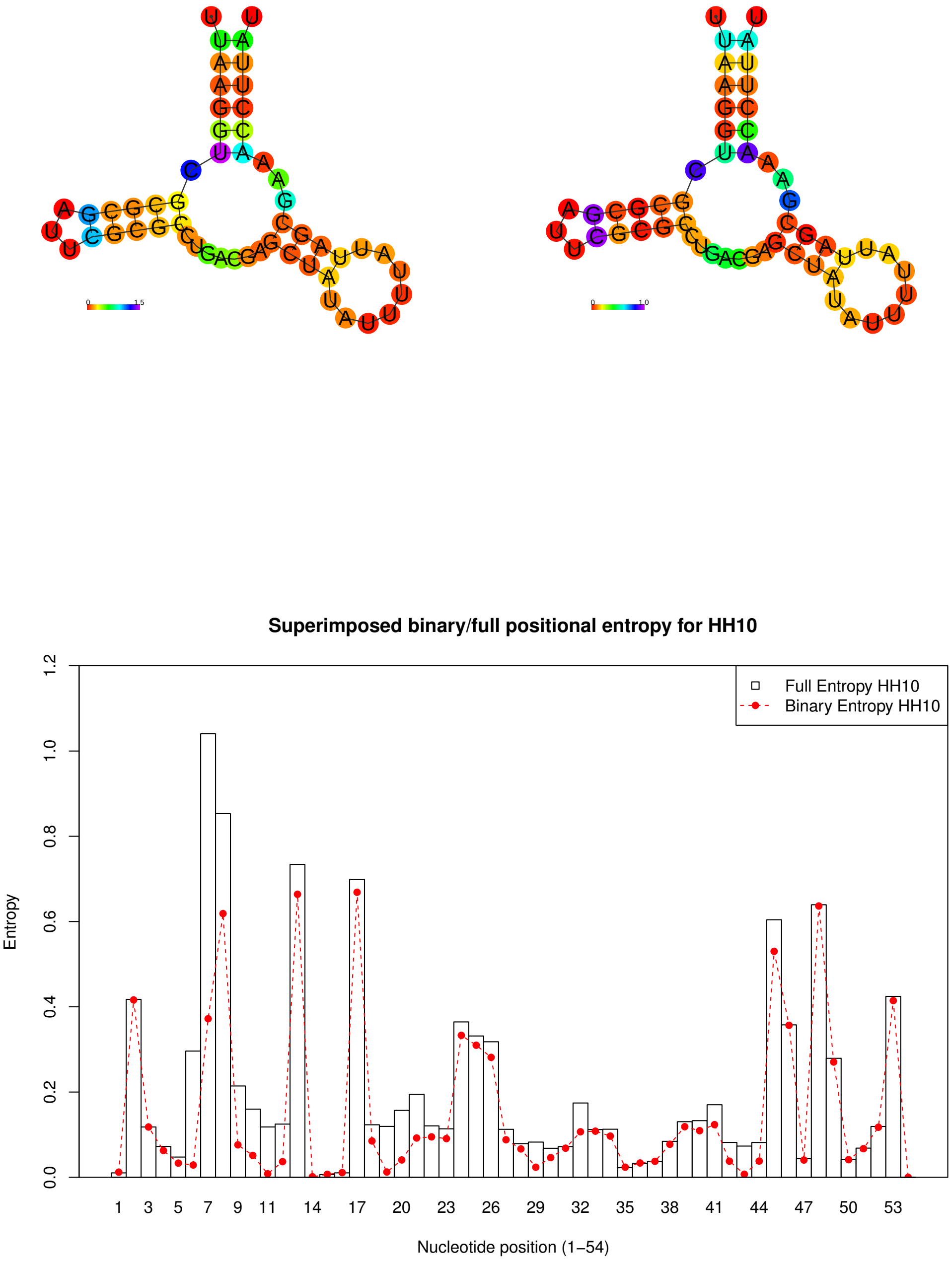

Figure 18: *(Left)* Full structural positional entropy of HH10. *(Right)* Binary structural positional entropy of HH10. *(Down)* Overlay of full and binary structural positional entropy of HH10.

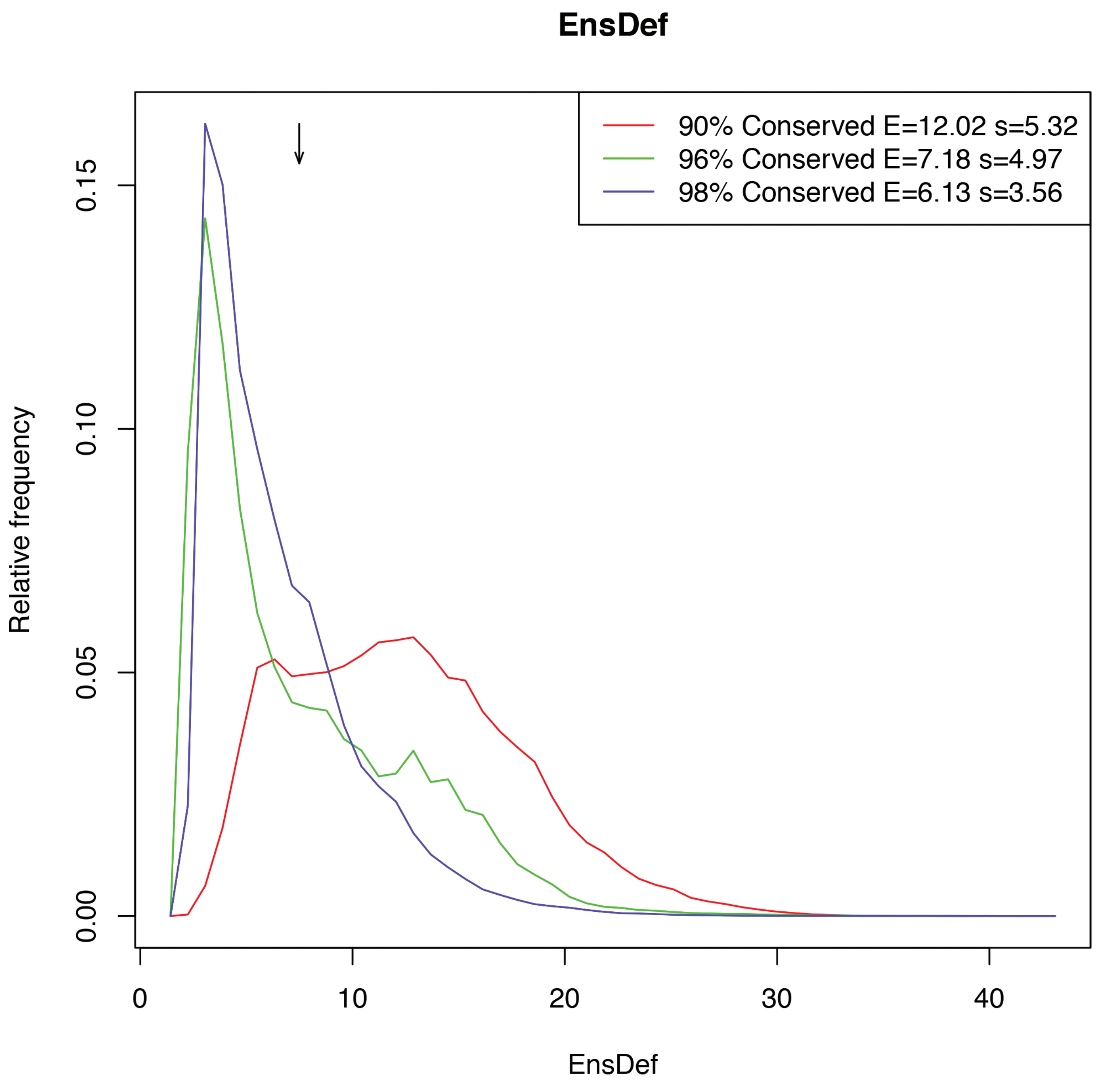

Figure 19: Ensemble defect for `RNAiFold` sequences at varying thresholds for sequence identity.

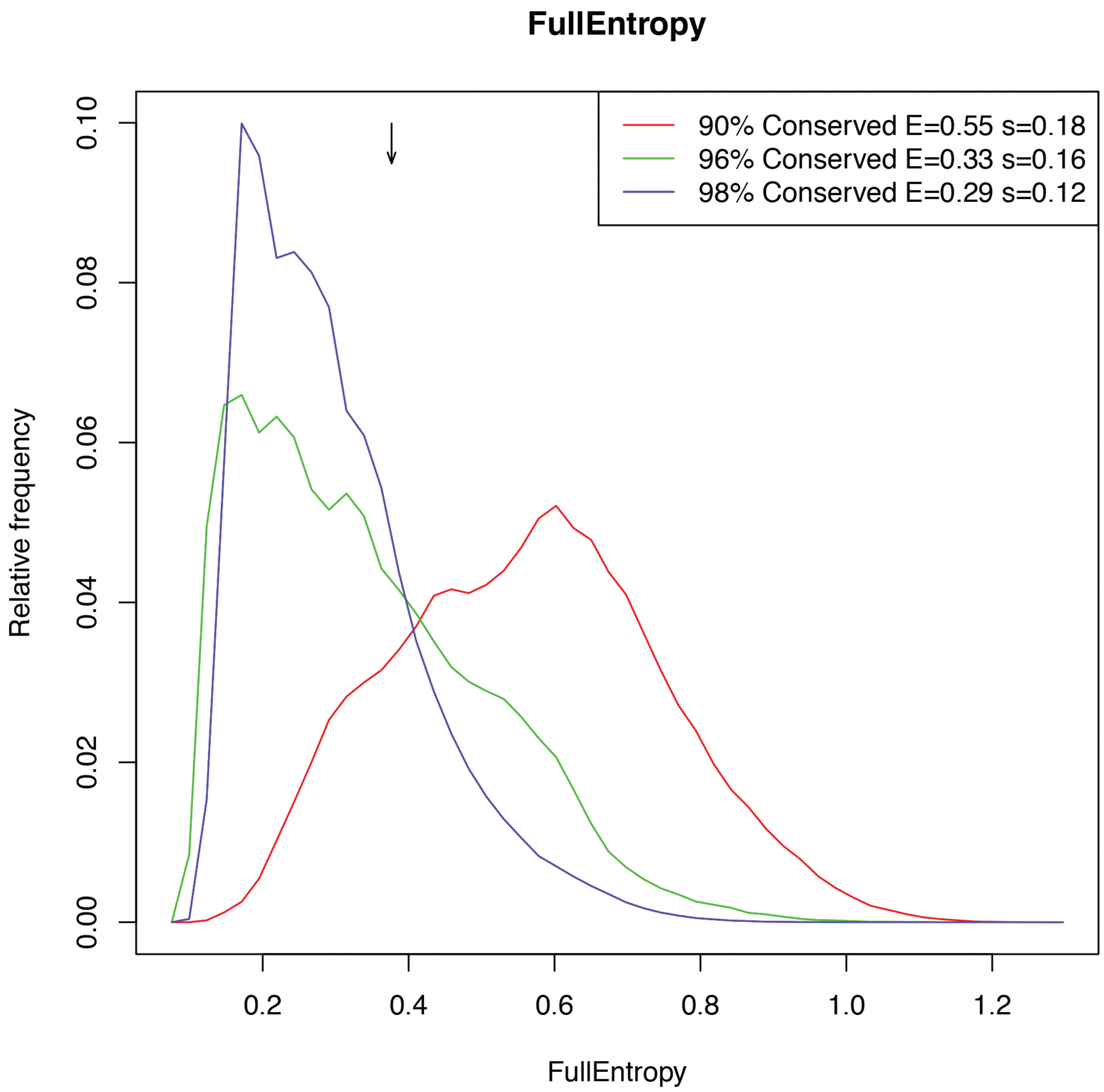

Figure 20: Full structural positional entropy for `RNAiFold` sequences at varying thresholds for sequence identity.

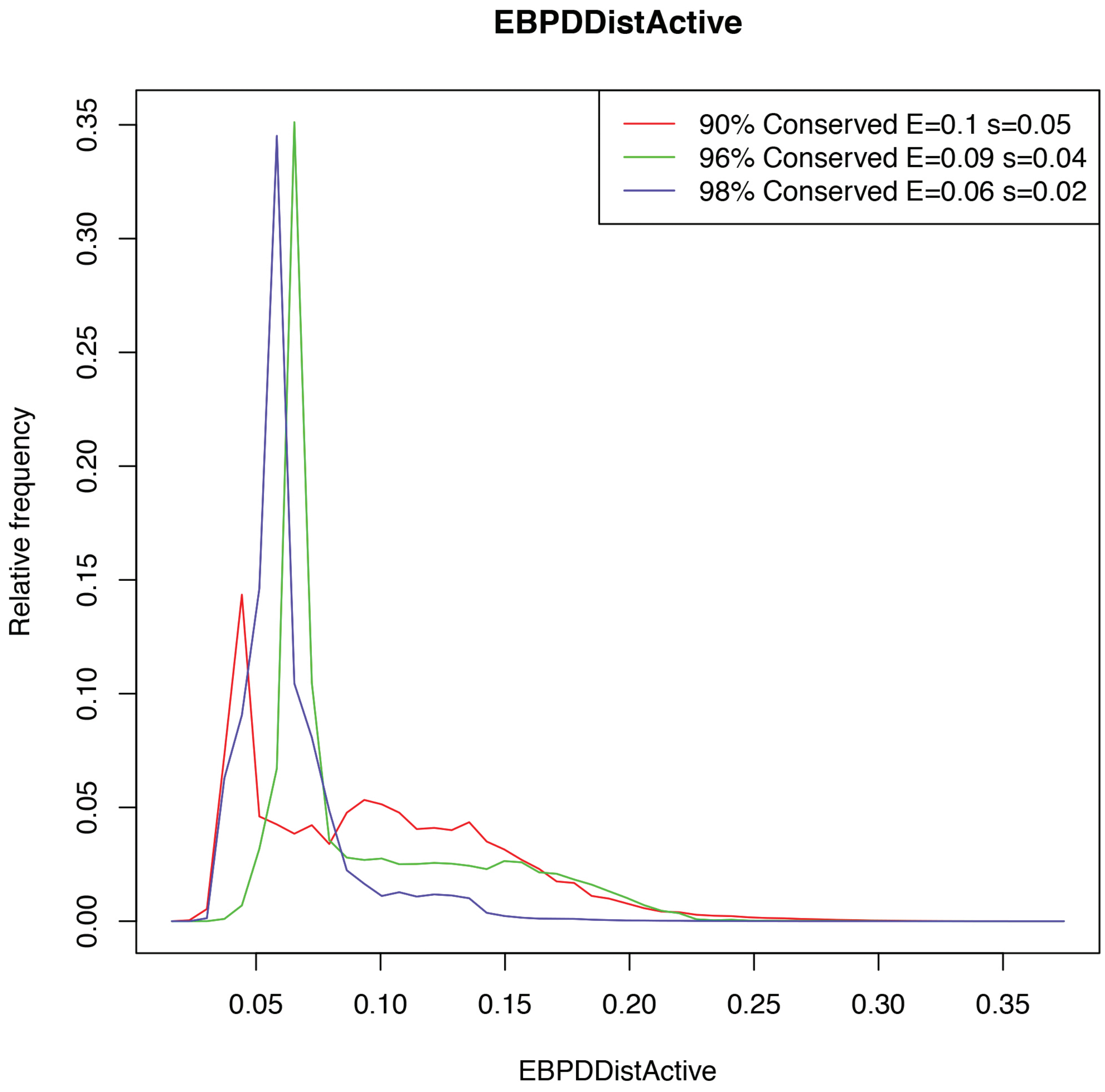

Figure 21: Expected base pair distance discrepancy for `RNAiFold` sequences, measured with respect to the 'active site' (i.e. the 15 constrained positions having 96% sequence identity in the Rfam seed alignment of PLMVd hammerhead).

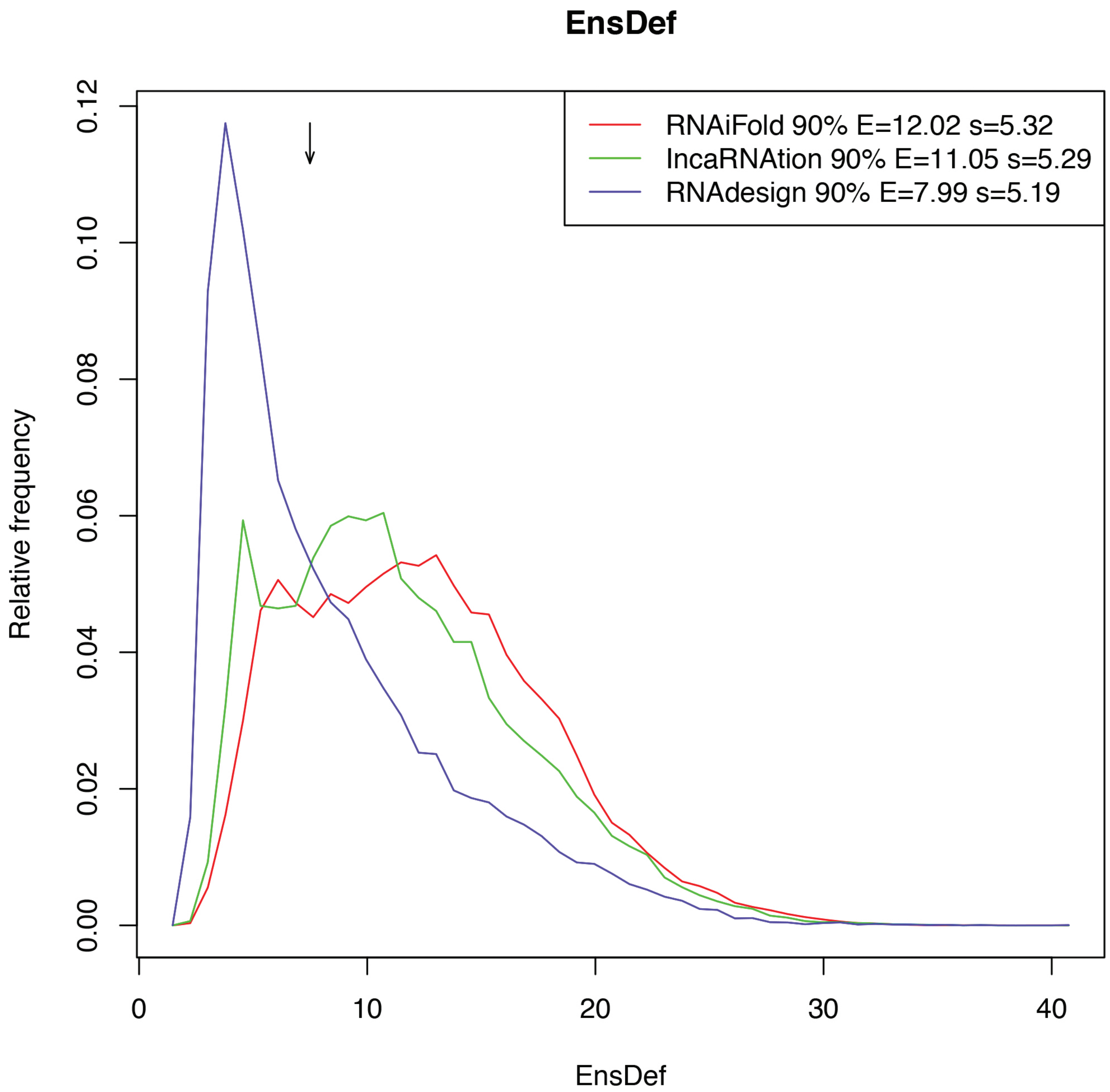

Figure 22: Comparison of ensemble defect for sequences from `RNAiFold`, `RNAdesign`, `IncaRNAtion`, when constraints are applied for positions exceeding 90% sequence identity, corresponding to fixing 19 nucleotides in the 54 nt sequences sought. Note that sequences returned by `RNAdesign` and `IncaRNAtion` had to be filtered to retain only those sequences, whose MFE structure matched the input target structure.

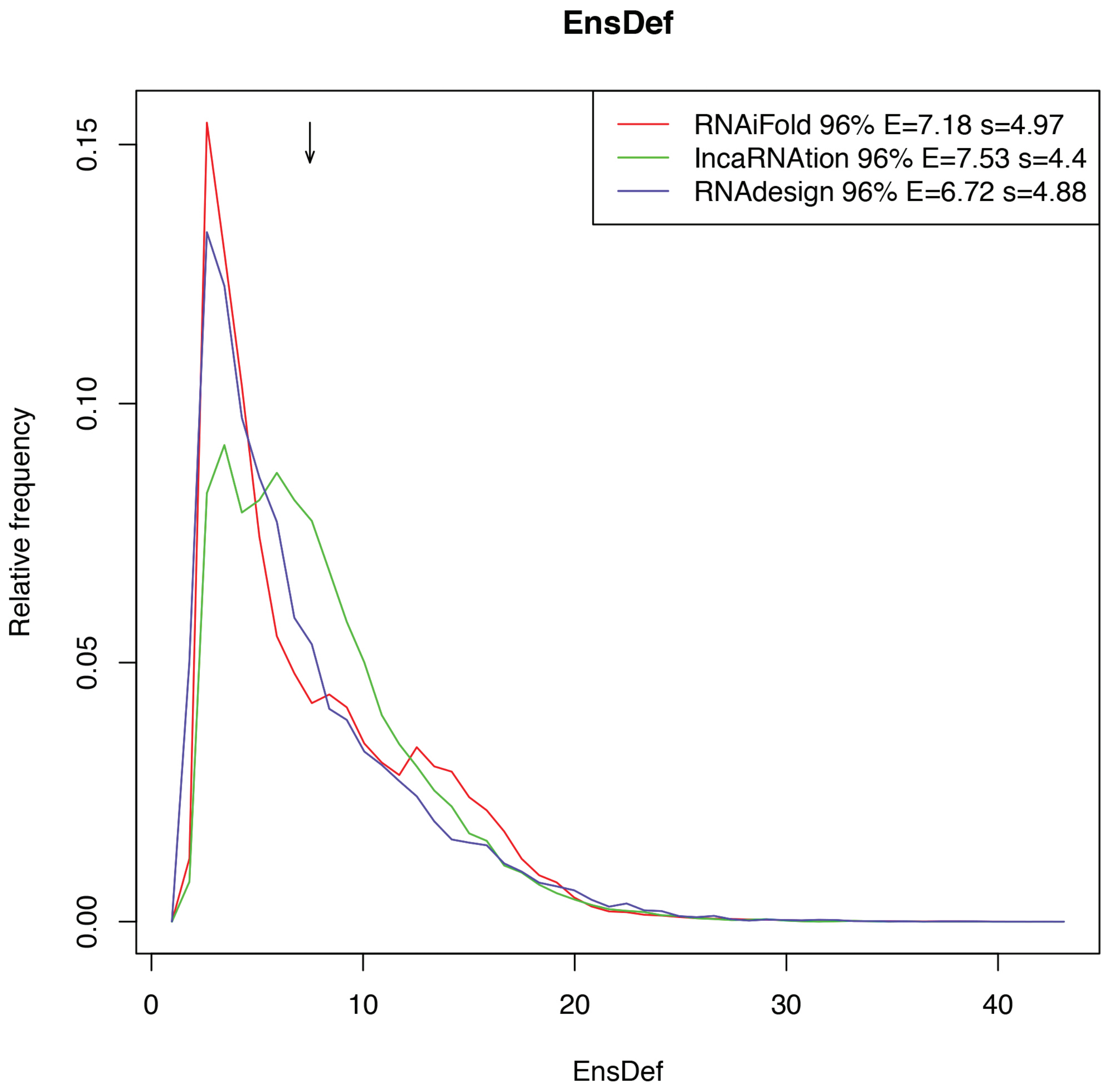

Figure 23: Comparison of ensemble defect for sequences from `RNAiFold`, `RNAdesign`, `IncaRNAtion`, when constraints are applied for positions exceeding 96% sequence identity, corresponding to fixing 15 nucleotides in the 54 nt sequences sought. Note that sequences returned by `RNAdesign` and `IncaRNAtion` had to be filtered to retain only those sequences, whose MFE structure matched the input target structure.

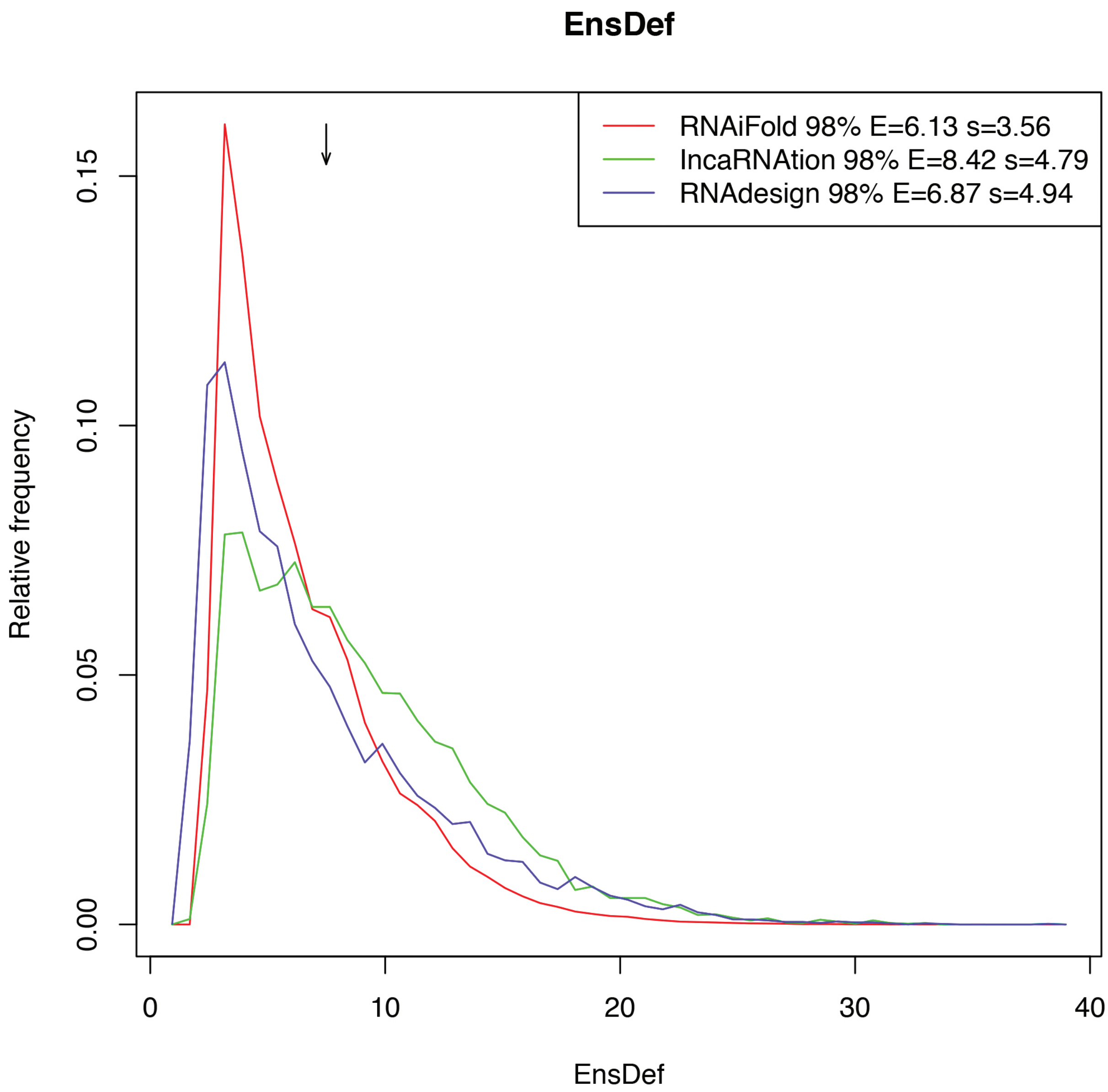

Figure 24: Comparison of ensemble defect for sequences from `RNAiFold`, `RNAdesign`, `IncaRNAtion`, when constraints are applied for positions exceeding 98% sequence identity, corresponding to fixing 11 nucleotides in the 54 nt sequences sought. Note that sequences returned by `RNAdesign` and `IncaRNAtion` had to be filtered to retain only those sequences, whose MFE structure matched the input target structure.
